# Supplementary material for: Structures of lipoprotein signal peptidase II from Staphylococcus aureus complexed with antibiotics globomycin and myxovirescin
Source: Nat Commun. 2020 Jan 9;11:140. doi: 10.1038/s41467-019-13724-y (PMC6952399; doi:10.1038/s41467-019-13724-y)
Supplement: Supplementary file 1 — Supplementary Information [file 41467_2019_13724_MOESM1_ESM.pdf]

Supplementary Information for

Structures of lipoprotein signal peptidase II from *Staphylococcus aureus* complexed with antibiotics globomycin and myxovirescin

Samir Olatunji[1,6], Xiaoxiao Yu[1,6], Jonathan Bailey[1,6], Chia-Ying Huang[2], Marta Zapotoczna[3], Katherine Bowen[4], Maja Remškar[5], Rolf Müller[5], Eoin M. Scanlan[4], Joan A. Geoghegan[3], Vincent Olieric[2], Martin Caffrey[1]*

* Correspondence and requests for materials should be addressed to M.C. (email: martin.caffrey@tcd.ie)

Supplementary Discussion
Supplementary Methods
Supplementary Figures 1-26
Supplementary Tables 1-5
Supplementary References

Supplementary Discussion

Comparison of LspMrs- and LspPae-globomycin complex structures

While the active and substrate binding sites are very similar in LspMrs and LspPae, there are two notable structural differences. The first is at the distal end of the β-cradle. In LspPae, both loops (L12, L34) connecting pairs of strands are upturned facing the extracytoplasmic space (Supplementary Fig. 15). In LspMrs, one loop faces away from while the other faces into the membrane. The different conformations reflect the polar residues (Gln131, Asn132, Arg133; LspPae numbering) that make up loop L34 in LspPae which have apolar counterparts (Ile124, Phe125, Gly126) in LspMrs. The second difference in structure is at the EL that sits directly above globomycin securing it in place (Supplementary Fig. 16). In LspMrs, the EL runs from Asn53 to Lys63. In LspPae, it is three residues longer extending from Asn54 to Gly67 (LspPae numbering). Both include a half-turn helix. The loop was shown in LspPae to be flexible and able to move laterally across the membrane surface[1]. In both orthologs, a conserved aromatic residue, Trp57 in LspMrs and Phe59 in LspPae, extends from the tip of the loop over globomycin clamping it against the surface of the substrate-binding pocket (Supplementary Fig. 16).

Both ortholog structures include six monoolein lipid molecules from the host mesophase used for crystallogenesis. For the most part, they recapitulate a membrane bilayer with individual molecules decorating the protein surface having head groups oriented toward the aqueous interface and acyl chains toward the membrane interior. Several occupy similar positions on the enzyme surface. Two from LspPae and one from LspMrs reside with head groups in the space between the end of H1 and the β-cradle. In both structures, a single monoolein molecule is situated with its head group facing into the active site and its acyl chain extending around H4 beneath strands 3 and 4 of the β-cradle (Supplementary Fig. 10). In both orthologs, the glycerol head group of this lipid coordinates with catalytic Asp118 and with residues in globomycin. We have speculated that this monoolein molecule takes the place of one of the chains and the head group in the DAG of the prolipoprotein substrate[1].

The surface electrostatics of LspMrs and LspPae show noted similarities and differences (Supplementary Fig. 17). The membrane interior and cytoplasmic facing surfaces that include the active site and substrate binding pockets are quite similar. A strongly negative region at the membrane interface just below the β-cradle corresponds to the catalytic dyad aspartates. However, the electrostatics of the EL and the cradle in the two orthologs are quite different. The cradle in LspMrs has a strongly negative surface while for LspPae the exposed cradle surface is a mix of negative, neutral and positive features. By contrast, the EL is more positive in LspMrs. Overall, the electrostatics of the extracytoplasmic surface is quite different in LspPae and LspMrs. LspPae is more neutral or mixed, LspMrs is considerably more negative.

These assorted structural and electrostatics differences undoubtedly account for the disparate specificities, activities and sensitivities of the two peptidases that must act on a multitude of different lipoprotein substrates. Such disparities might be exploited for rational antibiotic development.

Specific activity disparity between LspMrs and LspPae

The kinetic parameters, K_m and V_{max}, of LspMrs and LspPae differ in ways that suggest the former has the lower substrate affinity and turnover number. The FRET assays, upon which these parameters are based, were performed under conditions where the activity of each ortholog was optimized for reaction mixture composition and pH (see the Methods section and Supplementary Fig. 5). The gel-shift assay (Supplementary Fig. 4), which is a more complex coupled assay, does not lend itself to a full kinetic analysis. Nonetheless, the activity of LspMrs with proICP as substrate was 3-times less than that observed for LspPae under close to similar conditions (Supplementary Fig. 8, Supplementary Table 2). The purity of the enzymes used in the current study was comparable in both cases (Supplementary Fig. 2) and is not likely to account for such a difference in specific activity. Residual lipids that track with the protein through purification can influence activity. LspPae and LspMrs derive from Gram-negative and Gram-positive organisms, respectively, each with its own distinct membrane lipid profile. Notably, LspMrs from *S. aureus*, lacks PE but has lysyl-PG. By contrast, membranes from *P. aeruginosa* (and *E.*

coli) are rich in PE but lack lysyl-PG[2]. Thus, disparities in lipid content and identity, should they exist, may account for the observed activity difference. Similar differences in peptidase activity have been noted for LspA from *E. coli*, a Gram-negative bacteria, and from the Gram-positive bacteria *Streptococcus pyogenes*[3].

A shortcoming in the foregoing analysis concerns intrinsic substrate preference which may be different for the two LspA orthologs. Substrate optimization was not carried out in the current study. Thus, the gel-shift assay performed with both enzymes was done with recombinant proICP, a prolipoprotein substrate from *P. aeruginosa* (Supplementary Fig. 3a). Further, the lipopeptide substrate used for the FRET assays was based on the consensus sequence of residues on either side of the scissile bond in lipoproteins from *P. aeruginosa* (Supplementary Fig. 3b). Thus, neither substrate may be optimal for either enzyme and especially so for LspMrs thereby accounting for its lower specific activity. To understand the origins of the disparity, a substrate optimization study is needed. This would involve exploring such variables as lipobox sequence, number and sequence of residues on either side of the lipobox, DAG acyl chain length and modification types (such as number, position and isomeric type of unsaturation, branching, etc.) and FRET chromophore identity. Such a study is in progress but beyond the scope of the current investigation.

It is possible that the difference in specific activity is intrinsic to the two enzymes reflecting their respective amino acid sequences and molecular structures that have evolved in response to their distinctive cellular constitutions and the different environments in which they grow. LspPae and LspMrs have sequence identities and homologies of 31% and 52% (Supplementary Fig. 2), respectively, yet are structurally homologous with a C_α r.m.s.d. value over 155 residues of 0.713 Å. Surface electrostatics and structural differences at the distal end of the β–cradle and at the EL have been discussed (Supplementary Discussion) and these may well contribute to the disparity in specific activity and sensitivity to globomycin (Table 1).

FRET lipopeptide and prolipoprotein as substrates for LspA

LspA is a signal peptidase. It cleaves the N-terminal signal peptide from a prolipoprotein. In *P. aeruginosa*, these lipoproteins vary in size from about 50 to close to 1,000 residues. For the purpose of high-throughput screening of compound libraries, a single molecule FRET-based assay was developed. The substrate used in the assay was a diacylglyceryl-hexapeptide that included the consensus lipopbox sequence LAGC* from *P. aeruginosa* with fluorophore and quencher molecules at either end (Supplementary Fig. 3b). Despite its small size, this lipopeptide acted as a substrate for both LspPae and LspMrs (Supplementary Fig. 5). However, when it comes to translating hits identified with a FRET lipopeptide to leads for progression to a useful therapeutic, caution is needed. The FRET lipopeptide may not faithfully mimic natural lipoprotein substrates and, in turn, may provide false leads in drug discovery. A FRET lipopeptide can also provide unreliable information regarding the kinetics and mechanism of LspA as a signal peptidase. For these reasons, having in place a complementary assay that uses an actual lipoprotein substrate makes good sense. Such an assay was developed for LspA using proICP, a lipoprotein from *P. aeruginosa*. The assay relies upon the prior generation of proICP from pre-proICP which is dagylated by the first enzyme in the lipoprotein processing pathway, Lgt (Fig. 1). Separating the substrate, proICP, from its products, signal peptide and dagylated lipoprotein, by sodium dodecyl sulfate–polyacrylamide gel electrophoresis (SDS-PAGE) followed by Coomassie staining and digital imaging provides a means for quantitation, as implemented in this study (Supplementary Fig. 4). Specific activity values, recorded under as close to similar conditions as was possible, agree well for the two methods in assays of LspPae activity (Supplementary Table 2). However, in the case of LspMrs, the recorded specific activity was 20-fold lower by the FRET method. It would appear therefore that proICP is better than the lipopeptide as a substrate for LspMrs. When dose-response measurements were made for globomycin and myxovirescin, similar IC_{50} values approaching the enzyme concentration used for assay were recorded for LspPae by the two methods (Table 1 and Supplementary Table 3). However, for LspMrs, the two methods reported very different IC_{50} values with that for globomycin some three orders of magnitude higher at 171 μM with the proICP substrate compared to 0.17 μM with the FRET lipopeptide substrate (Table 1 and Supplementary Table 3, Supplementary Fig. 4e). These results highlight the dramatic differences between the sensitivities of LspMrs and LspPae to globomycin and

the different results that can be obtained by the two methods. For the bulk of this work therefore we refer, for the most part, to data obtained using assays with the natural lipoprotein substrate in gel-shift assays (Table 1).

One consequence of LspMrs having a relatively low specific activity is that, in order to get a reliable signal in activity assays, it was necessary to raise enzyme concentration. For kinetic measurements generally, it is desirable to use an enzyme concentration that is much lower than that of the substrate. In this situation, the usual assumption that added and free substrate concentrations are equal is valid. At high enzyme concentrations [E] therefore, and especially in the case of tight ligand binding where the inhibition constant, K_i, is small, a measured IC_{50} is limited to [E]/2 [4–6]. In such circumstances, a meaningful IC_{50} or K_i can only be obtained with considerable effort. With a few exceptions in the current study, this situation prevailed with both LspA orthologs where IC_{50} values came close to [E]/2 (Table 1 and Supplementary Table 3, Supplementary Fig. 6). The exceptions arose with LspMrs when assays were performed with the proICP substrate in the presence of globomycin. The wild-type variant of this ortholog binds weakly to globomycin with an IC_{50} of 171 μM when the assay was performed at an enzyme concentration of 0.5 μM. In this case, a reliable apparent inhibition constant, K_i', can be obtained and a K_i estimated provided the type of inhibition is known.

Inhibition type

Inhibition of LspA from *E. coli* by globomycin has been described as non-competitive[7]. The evidence provided in support of this conclusion included an insensitivity of IC_{50} to substrate concentration and the fact that the V_{max} but not the K_m changed with globomycin concentration. It was of interest to determine if a similar mode of inhibition applied to LspPae and LspMrs. From published work[7–9] and from FRET-based kinetics measurements reported here, we know that globomycin binds with high affinity (Supplementary Fig. 6). Tight binding means that a more in-depth analysis must be carried out in order to identify inhibition type. For such analysis, it is important to know active enzyme concentration accurately. Fortunately, it was possible to use the property of tight inhibitor binding to measure the concentration of active enzyme (Supplementary Fig. 18a). In the case of LspPae, the estimated value and the concentration determined spectroscopically agreed remarkably well. Parenthetically we note that knowing active LspPae concentration accurately enabled a characterization of the myxovirescin sample used in this study. Myxovirescin also binds tightly to LspA (Supplementary Fig. 6). However, it was available in very small amounts and there was a concern about its actual concentration. Titrating LspPae with myxovirescin established that the material supplied was 0.73-fold its nominal concentration (Supplementary Fig. 18, b and c). The corrected value was used in all subsequent work in this study.

To determine inhibition type in the case of a tight binding inhibitor, measurements should be performed ideally where [E] is well below the K_i'. Because LspMrs has a low specific activity, kinetic measurements of the type needed to explore inhibition mode were not possible with the current FRET assay when conducted at such low enzyme concentrations. Our attention therefore turned to LspPae, and Morrison analysis was used to estimate K_i' for globomycin and myxovirescin (Supplementary Fig. 18). This involved fitting fractional velocity (v_i/v_o) data plotted as a function of inhibitor concentration [I] with the Morrison equation[5,10]:

$$v_i / v_o = 1 - (([E] + [I] + K_i') - (([E] + [I] + K_i')^2 - 4[E][I])^{1/2}) / 2[E] \qquad (1)$$

Since [E] and [I] are known and v_o and v_i are measured, K_i', the only unknown can be obtained from a best fit to the data. The analysis yielded estimated K_i' values of 740 ± 410 and 47.6 ± 128 pM for globomycin and myxovirescin, respectively, consistent with tight binding.

Ultimately, we seek to evaluate K_i for each inhibitor. To do so, it is necessary to know the inhibition type and to use the corresponding equation relating IC_{50} and K_i. This can be done by establishing how IC_{50} values scale with substrate concentration [S]. For tight binding non-competitive inhibition, IC_{50} is independent of [S] and is directly proportional to K_i:

$$IC_{50} = K_i + [E]/2 \qquad (2)$$

For tight binding competitive inhibition, the dependence of IC_{50} on [S] is linear

$$IC_{50} = K_i' + [E]/2 = K_i(1 + [S]/K_m) + [E]/2 \quad (3)$$

In the case of tight binding uncompetitive inhibition, IC_{50} depends on [S] by an inverse power:

$$IC_{50} = K_i' + [E]/2 = K_i(1 + K_m/[S]) + [E]/2 \quad (4)$$

The lowest LspPae concentration that gave reliable FRET signals and initial velocity data was 20 nM. The relevant measurements, conducted at this concentration, involved determining how IC_{50} scales with substrate concentration, [S]. The data are shown in Supplementary Fig. 19. Unfortunately, due to the tightness of globomycin and myxovirescin binding, the signal-to-noise in the data was insufficient to reliably identify inhibition type. Clearly, assays must be run at significantly lower enzyme and wider substrate concentrations than used here and with better signal-to-noise ratio to definitively identify inhibition mode.

Rationalizing non-competitive inhibition

The reaction scheme for an enzyme interacting with an inhibitor (Supplementary Fig. 20) has three major modes of inhibition: competitive, non-competitive and uncompetitive. In competitive inhibition, the inhibitor interacts with the free enzyme. An uncompetitive inhibitor by contrast, interacts with the enzyme-substrate complex. Under non-competitive inhibition conditions, the inhibitor can react with the free enzyme and the enzyme-substrate complex, and the substrate can bind with the enzyme-inhibitor complex. Because we can form LspA-globomycin and LspA-myxovirescin (EI) complexes (for crystal structure determination), neither antibiotic can be an uncompetitive inhibitor, which binds only to the binary ES complex. However, both competitive and non-competitive inhibition modes allow for EI formation and thus both are possible inhibition types for globomycin and myxovirescin interacting with LspPae. A more sensitive assay is being developed to resolve the lingering ambiguity.

Competitive inhibition of LspA is easily understood in light of the current crystal structures of the enzyme in complex individually with two antibiotics. However, in the case of non-competitive inhibition, the ligand can exist as a ternary complex (Supplementary Fig. 21) with the enzyme and the substrate. How such a complex might form is less obvious and is worth considering in the context of the solved crystal structures.

The prolipoprotein substrate of LspA can be viewed as consisting of four recognizable parts (Supplementary Fig. 21). These include the cleavage site in which resides the scissile bond, the signal peptide (SP), the protein (P) and the DAG. The enzyme has four docking sites, one each for the cleavage site, the SP, the P and the DAG. Given the flexible nature of the linkages between the different parts of the substrate, it seems reasonable that it can engage with the enzyme via one or more docking sites at any one time. But only when the three peripheral parts are docked properly is the scissile bond positioned in the active site for Michaelis complex formation and for cleavage. A non-competitive inhibitor can bind to the free enzyme, E, and to the enzyme to which the substrate has attached with one or more of its peripheral docking sites, ES. In this simplified model that does not accommodate allosteric binding, a non-competitive inhibitor cannot bind to the enzyme with all three peripheral sites and the active site occupied with substrate simultaneously. This means there are at least six inhibitor-free and six inhibitor-bound states with substrate docked at one or two sites, as schematized in Supplementary Fig. 21.

Given the solved structures and the flexibility of the connections between the various parts of the substrate, it makes sense that docking of the type proposed could occur. This is particularly true in the case of single site docking where the DAG, the SP or the P parts would engage with the respective docking sites on the enzyme as discrete entities. Two-site docking might be a little more difficult to rationalize but with flexible linkers in the prolipoprotein such two-point docking is not unreasonable. However, the two-point docked state with the SP and P parts docked would likely block the active site and a large part of the binding pocket within the membrane and thus prevent inhibitor binding. The model for pure non-competitive inhibition requires that the one- and two-point docked states do not preclude inhibitor binding to the active site. Therefore, this particular SP+P docked state may not make sense in light of our current thinking of how the substrate engages with the enzyme as a Michaelis complex. The model just described suggests the possibility of capturing a structure of the enzyme in

complex with the inhibitor and the substrate simultaneously. However, given the multitude of states that could possibly exist when substrate and inhibitor are present, crystallization may be challenging. Single particle cryo-electron microscopy may prove more informative in characterizing these assorted intermediate states.

Complex nature of the LspA assay reaction mix

LspA uses a prolipoprotein as a substrate and it generates a signal peptide and an apolipoprotein as products (Fig. 1). The enzyme, the prolipoprotein and the signal peptide are all either integral membrane proteins or peptides while the apolipoprotein is membrane anchored via a diacylglyceryl moiety. It is necessary to quantify the activity of an enzyme to establish its kinetics and mechanistic enzymology, its specificity and for compound library screening in the case of it being a drug target. Ideally, an activity assay is simple to perform, is homogenous in that it can be carried out in a single reaction vessel, uses very little material and lends itself to high-throughput applications. It must also, as much as possible, report faithfully on the properties of the enzyme in its native environment. In the case of LspA, it originates from the cytoplasmic membrane of a bacterial cell.

In this study, we have used two types of activity assay. The first is a coupled assay that employs a native or close to native prolipoprotein substrate. The particular prolipoprotein used is ICP from *P. aeruginosa*. The precursor form of the substrate, pre-proICP, with an N-terminal hexahistidine tag, is expressed recombinantly in *E. coli*. It is then converted to proICP by the action of a purified recombinant Lgt. Lgt catalyzes the formation of a thioether linkage between a diacyglyceryl group derived from 1,2-dioleoylphosphatidylglycerol (DOPG) and the thiol group of cysteine in the lipobox of pre-proICP. The assay is typically run by first generating the proICP in a reaction that lasts one hour. To this reaction mix that contains freshly made proICP is added LspA. The peptidase reaction is allowed to progress for a fixed period and is then stopped by adding SDS. Quantitation involves SDS-PAGE analysis of the reaction mix to ideally separate the proICP, signal peptide and apoICP from one another and from other proteins in the system that include Lgt, LspA and residual pre-proICP. Coomassie staining of the gel followed by digital imaging is used for quantitation (Supplementary Fig. 4).

Complications arise when proteins do not electrophorese as single bands and when bands overlap. In this regard, one would expect to benefit from the general practice of using enzymes at catalytic, and thus very low concentrations. Unfortunately, this was not possible in the current assay, for Lgt at least, where its concentration was ramped up to boost the level of proICP generated which served as a substrate for LspA. Another complication has to do with the fact that the Lgt reaction is not stopped before initiating the LspA reaction. This means Lgt may continue to generate proICP at an unknown rate during the course of the subsequent LspA assay. Each of the proteins in this assay system requires solubilizing in detergents. In separate studies, the optimal detergent was chosen for each individually based usually on yield and homogeneity as judged by size-exclusion chromatography and SDS-PAGE. Thus, Lgt and pre-proICP uses lauryl maltose neopentyl glycol (LMNG) detergent at pH 7.5, LspMrs uses *n*-dodecyl β-D-maltoside (DDM) detergent at pH 6.2 and LspPae uses *n*-dodecyl phosphocholine detergent at pH 6.2. In a given assay, these assorted ingredients are combined with undetermined consequences in terms of activity, solubility, and state of dispersion. The products of the LspA reaction, signal peptide and apoICP, have their own physicochemical properties that may or may not be matched to those provided in the reaction mix. This, in turn, could impact on rates of reaction. Likewise, the antibiotics used in this study have properties that need to be considered in the context of IC_{50}, K_i' and K_i determinations. The assumption is that the ligand remains as a monomer throughout the investigation where added concentration corresponds to the concentration of active species, presumably the monomer, interacting with the enzyme. However, if these ligands partition selectively at interfaces or into subsets of mixed micelles or they self-associate, then an assumed activity coefficient of one is inappropriate.

This lipoprotein-based assay system is obviously a complex one. But it works. However, it is slow, tedious to perform and it requires a number of specialized ingredients that are not commercially available. Fortunately, it can be carried out with the Lgt and LspA reactions run sequentially in the same vessel. It means however that the proICP substrate for LspA is present in amounts that are not easily quantified and it comes with several other ingredients that are part of the Lgt reaction mix including the small molecule product, glycerol 1-phosphate. The fact that the proICP substrate concentration is not

known accurately means that quantitative kinetics and mechanistic studies on LspA cannot be performed easily. Nonetheless, it has been possible to use the assay to estimate specific activity values for the LspMrs and LspPae orthologs, to monitor the impact of site directed mutations on activity and to make IC_{50} measurements for two antibiotics (Table 1). Importantly, with proICP as a substrate the results obtained are likely to reflect the properties of the enzyme acting on a close to native substrate.

A single molecule FRET-based assay is the second method used to quantify LspA peptidase activity. Several peptides were tested that included a hexamer and an octamer. In all cases, aminobenzoic acid at the N-terminus and nitro-tyrosine at the C-terminus were used as fluorophores and quenchers, respectively. Dipalmitin in thioether linkage to the thiol of cysteine in the lipobox was employed as the diacylglyceryl moiety. The FRET lipopeptide used in this study is a hexapeptide based on the *P. aeruginosa* consensus sequence with the enantiomerically pure R-form of dipalmitin (Supplementary Fig. 3b).

The FRET-based assay was designed for high-throughput applications. It can be run routinely in 96- and 384-well plates. The assay is typically performed by pre-incubating all ingredients except the enzyme and then starting the reaction by adding LspA. Fluorescence is monitored at 37 °C over a period of time providing a progress curve from which the initial reaction rate is calculated. Ideally, rate is constant from time zero and drops as substrate is consumed. Under certain circumstances however we have found that the rate accelerates over the first few minutes before reaching a constant value. This shows up as an initial lag in the progress curve. Lags can arise for reasons that include thermal equilibration, physicochemical equilibration, substrate inhibition, product activation, contamination with non-FRET substrate and tardy changes in the enzyme’s conformation as it adjusts to the reaction mix that may contain one or more new detergents, a substrate and/or an inhibitor. Physicochemical equilibration refers mostly to solubility issues and to detergent mixing and equilibration. This is an important factor in the current assay system since the solubility of all components that include the enzyme, the substrate, the two products and the antibiotic inhibitors all rely on detergents to keep them in solution and ideally in a monomeric state. The origin of the lag observed in this work was not investigated. It would constitute a study in itself. In all cases, initial rate measurements were made after the lag in the progress curve was complete and a constant rate had been established.

Supplementary Methods

FRET lipopeptide synthesis and purification

Proton nuclear magnetic resonance (^{1}H NMR) and carbon nuclear magnetic resonance (^{13}C NMR) spectra were recorded on a Bruker Advance 400 spectrometer, ^{1}H (400.13 MHz) and ^{13}C (100.6 MHz) or a Bruker Ultrashield 600 spectrometer, ^{1}H (600.13 MHz) and ^{13}C (150.6 MHz). Resonances δ, are in parts per million calibrated using residual undeuterated solvent (^{1}H NMR) or the deuterated solvent (^{13}C NMR) as internal reference standards. Infrared spectra (IR) were recorded on a Perkin Elmer spectrometer. Mass spectrometry analysis was performed with a Q-Tof Premier Waters Maldi-quadrupole time-of-flight (Q-Tof) mass spectrometer equipped with Z–spray electrospray ionization (ESI) and matrix assisted laser desorption ionization (MALDI) sources. Silica gel Florisil (200 mesh; Aldrich) was used for column chromatography. Thin-layer chromatography (TLC) was performed using Merck 60 F254 silica gel (pre-coated, 0.2 mm thick, 20 × 20 cm) and visualised by UV (λ_{max} = 254 nm), ammonium molybdate, permanganate or ninhydrin staining. Other reagents were purchased from an industrial supplier. In the following section, the FRET peptide and intermediates used in its synthesis are identified in shorthand by emboldened arabic numerals.

Peptide synthesis

(3*S*,6*S*,12*R*,16*R*)-12-(((*S*)-1-(((*S*)-1-(((*S*)-1-amino-3-(4-hydroxy-3-nitrophenyl)-1-oxopropan-2-yl)amino)-3-hydroxy-1-oxopropan-2-yl)amino)-3-hydroxy-1-oxopropan-2-yl)carbamoyl)-1-(2-aminophenyl)-3-isobutyl-6-methyl-1,4,7,10-tetraoxo-14-thia-2,5,8,11-tetraazaheptadecane-16,17-diyl dipalmitate - H-Abz-Leu-Ala-Gly-Cys(Pam)$_2$-Ser-Ser-Tyr(3-NO$_2$)-CONH$_2$ **(1)**

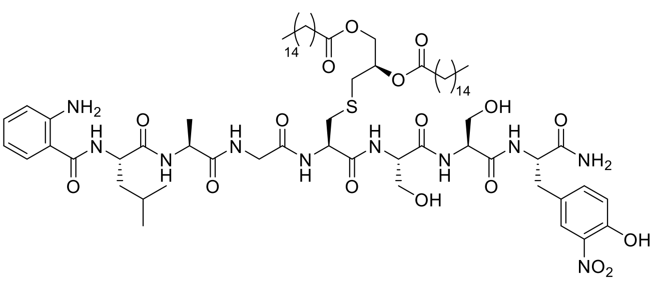

Peptide **1** is the FRET lipopeptide substrate used for assaying the peptidase activity of LspA. It was synthesised using manual Fmoc/*t*Bu solid phase peptide synthesis performed in polypropylene syringe reaction vessels (10 mL; Torviq, MI, USA). All reactions were performed at room temperature under continuous agitation. Rink amide (aminomethyl)polystyrene resin (286 mg, 0.20 mmol) with an initial loading of 0.70 mmol/g was loaded into the syringe and swollen in DMF (6 mL) for 1 h under agitation. The resin bound Fmoc was deprotected using 20 %(v/v) piperidine in DMF (2 × 10 min; 5 mL) and the deprotected resin was washed with DMF (3 × 5 mL), CH_2Cl_2 (3 × 5 mL) then DMF (3 × 5 mL). Coupling of the first amino acid to the resin was performed using PyBOP (4 equiv, 416 mg, 0.80 mmol), NMM (8 equiv, 0.175 mL, 1.60 mmol) and Fmoc-Tyr(3-NO$_2$)-OH (**6**) (4 equiv, 358 mg, 0.80 mmol) in DMF (4 mL). This solution was transferred to the resin in the syringe and agitated for 45 min. Excess reagents were drained from the reaction vessel and the resin was washed with DMF (3 × 5 mL), CH_2Cl_2 (3 × 5 mL) and DMF a second time (3 × 5 mL). Sequential coupling of Fmoc-Ser(*t*Bu)-OH (× 2) (3 equiv, 230 mg, 0.60 mmol), Fmoc-Cys(Pam)$_2$-OH (**5**) (3 equiv, 536 mg, 0.60 mmol), Fmoc-Gly-OH (3 equiv, 178 mg, 0.60 mmol), Fmoc-Ala-OH (3 equiv, 212 mg, 0.60 mmol), Fmoc-Leu-OH (3 equiv, 187 mg, 0.60 mmol) and Boc-Abz-OH (**7**) (3 equiv, 142 mg, 0.60 mmol) was performed using coupling cycles consisting of (i) Fmoc deprotection using 20 %(v/v) piperidine in DMF (2 × 10 min; 5 mL), (ii) resin washing with DMF (3 × 5 mL), CH_2Cl_2 (3 × 5 mL) then DMF (3 × 5 mL), (iii) peptide coupling with addition of PyBOP (3 equiv, 312 mg, 0.6 mmol), NMM (6 equiv, 0.132 mL, 1.2 mmol) and Fmoc-amino acid (3 equiv; 0.2 M) in DMF to the peptide resin for 45 min, (iv) resin washing with DMF (3 × 5 mL), CH_2Cl_2 (3 × 5 mL) then DMF (3 × 5 mL). Following the final coupling, the resin was treated with 20 %(v/v) piperidine in DMF (2 × 15 min; 5 mL) and the resin was washed with DMF (3 ×

5 mL), CH_2Cl_2 (3 × 5 mL), DMF (3 × 5 mL) and CH_2Cl_2 (3 × 5 mL), followed by drying of the resin under reduced pressure. The dry peptide resin was swollen in CH_2Cl_2 (5 mL) under agitation for 1 h, then drained. The cleavage cocktail (TFA:TES:H_2O; 95:2.5:2.5; 5 mL) was added to the syringe which was agitated for 90 min. The cleavage cocktail was drained and collected. The resin was washed with cleavage cocktail (2 × 2.5 mL) and the combined solution was concentrated *in vacuo* followed by precipitation of the peptide with Et_2O (10 mL) at 0 °C. The crude peptide collected by centrifugation and washed with Et_2O (2 × 10 mL) at 0 °C. The crude material was dried *in vacuo* and the peptide was purified by silica column chromatography (CH_2Cl_2 – CH_2Cl_2:MeOH 19:1) to yield a yellow solid (29.7 mg, 11%).

TLC (CH_2Cl_2:MeOH 9:1) R_f = 0.32; ^{1}H NMR (600 MHz, DMSO-d_6) δ 8.22 (d, *J* = 7.5 Hz, 1H, Cys-Ser-NH), 8.13 (d, *J* = 8.0 Hz, 1H, Leu-NH), 8.11 – 8.06 (m, 2H, Gly-NH, Cys-NH), 8.04 (d, *J* = 7.2 Hz, 1H, Tyr-Ser-NH), 8.02 (d, *J* = 7.2 Hz, 1H, Ala-NH), 7.93 (d, *J* = 8.1 Hz, 1H, Tyr-NH), 7.73 – 7.69 (m, 1H, Tyr-Ar-CH), 7.55 (d, *J* = 7.5 Hz, 1H, Abz-Ar-CH), 7.36 (d, 1H, *J* = 8.0 Hz, 1H, Tyr-Ar-CH), 7.27 – 7.21 (m, 2H, $CONH_2$), 7.13 (t, *J* = 8.2 Hz, 1H, Abz-Ar-CH), 6.98 (d, *J* = 8.0 Hz, 1H, Tyr-Ar-CH), 6.69 (d, *J* = 8.2 Hz, 1H, Abz-Ar-CH), 6.54 – 6.49 (m, 1H, Abz-Ar-CH), 6.35 – 6.31 (m, 2H, Abz-NH_2), 5.20 – 5.15 (m, 1H, Cys-Ser-OH), 5.12 – 5.07 (m, 1H, *S*-glyceryl-CH), 4.96 (t, *J* = 5.5 1H, Tyr-Ser-OH), 4.57 – 4.52 (m, 1H, Cys-αCH), 4.50 – 4.45 (m, 1H, Leu-αCH), 4.39 – 4.33 (m, 2H, Cys-Ser-αCH, Tyr-αCH) 4.31 – 4.26 (m, 2H, Ala-αCH, *S*-glyceryl-*O*CH_aH_b), 4.22 – 4.18 (m, 1H, Tyr-Ser-αCH), 4.11 – 4.05 (m, 1H, *S*-glyceryl-*O*CH_aH_b), 3.80 – 3.71 (m, 2H, Gly-CH_2), 3.66 – 3.60 (m, 1H, Cys-Ser-CH_aH_b), 3.59 – 3.52 (m, 2H Cys-Ser-CH_aH_b, Tyr-Ser-CH_aH_b), 3.52 – 3.47 (m, 1H, Tyr-Ser-CH_aH_b), 3.04 – 2.98 (m, 1H, Tyr-CH_aH_b), 2.91 (dd, *J* = 13.5, 4.6 Hz, 1H, Cys-CH_aH_b), 2.82 (dd, *J* = 14.0, 5.4 Hz, 1H, *S*-glyceryl-CH_aH_b), 2.76 – 2.71 (m, 1H, Tyr-CH_aH_b), 2.70 – 2.65 (m, 1H, *S*-glyceryl-CH_aH_b), 2.63 – 2.61 (m, 1H, Cys-CH_aH_b) 2.30 – 2.21 (m, 4H, Pal-αCH_2 x2), 1.71 – 1.62 (m, 3H, , Leu-CH_2, Leu-CH-CH_3), 1.56 – 1.45 (m, 7H, Pal-CH_2 x2, Ala-CH_3,), 1.31 – 1.18 (m, 48H, Pal-CH_2), 0.93 – 0.81 (m, 12H, Leu-CH_3 x2, Pal-CH_3 x2) ppm; ^{13}C NMR (151 MHz, DMSO-d_6) δ 173.0 (Pal C=O), 173.0 (Pal C=O), 172.9 (CONH), 172.7 (Leu C=O), 172.7 (Ala C=O), 170.7 (Cys-Ser C=O), 170.6 (Cys C=O), 170.1 (Tyr-Ser C=O), 169.3 (Abz C=O), 169.2 (Gly C=O), 150.1 (Abz-qC-NH_2), 136.7 (Tyr-Ar-CH), 136.5 (Tyr-Ar-CH) , 132.3 (Abz-Ar-CH), 129.0 (Abz-Ar-CH), 125.8 (Tyr-Ar-CH), 116.7 (Abz-Ar-CH), 115.0 (Abz-Ar-CH), 114.9 (Abz-qC), 70.3 (*S*-glyceryl-CH), 64.0 (*S*-glyceryl-*O*CH_2), 62.1 (Cys-Ser-βCH_2), 61.8 (Tyr-Ser-βCH_2), 56.1 (Tyr-Ser-αCH), 55.3 (Cys-Ser-αCH), 54.3 (Tyr-αCH), 52.6 (Cys-αCH), 51.7 (Leu-αCH), 48.7 (Ala-αCH), 42.3 (Gly-CH_2), 36.3 (Tyr-βCH_2), 34.6 (Cys-βCH_2), 34.0 (Pal-αCH_2), 33.9 (Pal-αCH_2), 32.1 (Pal-CH_2), 31.8 (*S*-glyceryl-CH_2), 29.5, 29.4, 29.5, 29.3, 29.2, 29.2, 28.9 (Pal-CH_2), 25.0 (Leu-CH), 24.9 (Pal-CH_2), 24.9, 23.7 (Leu-CH_3), 22.6 (Pal-CH_2), 21.8 (Leu-CH_3), 18.7 (Ala-CH_3), 14.4 (Pal-CH_3) ppm; *m/z* HRMS ($MALDI^+$) calculated. $C_{71}H_{116}N_{10}O_{17}SNa$ = 1435.8138 $(M + Na)^+$. Found = 1435.8124; IR ν_{max} (thin film)/cm^{-1} 3283 (NH), 2924 (CH_2), 2852 (CH_2), 1746, 1631 (Amide I: CO, CN), 1536 (Amide II: CN, NH), 1369 (Amide III: CN, CO, CC), 1210 (CO).

Synthesis of Fmoc-Cys(Pam)$_2$-OH

Compounds **3**, **4** and **5** were synthesized according to a slightly modified literature procedure as shown in Supplementary Fig. 22. Spectral data were in good agreement with the literature[11].

tert-Butyl-*N*-(((9*H*-fluoren-9-yl)methoxy)carbonyl)-*S*-((*R*)-2,3-dihydroxypropyl)-L-cysteinate **(3)**

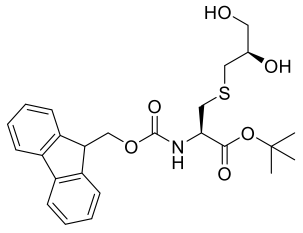

To a solution of (Fmoc-Cys-O*t*Bu)$_2$ (**2**) (3.50 g, 4.40 mmol) in anhydrous THF (20 mL) under argon was added PBu_3 (1.09 mL, 4.40 mmol). The reaction was stirred at room temperature for 5 min followed by

the addition of H_2O (4 mL). The solution was stirred at room temperature for 4 h which time TLC analysis indicated complete consumption of disulfide (R_f = 0.31; EtOAc:n-hexane 1:4) and formation of thiol (R_f = 0.46; EtOAc:n-hexane 1:4). The solvents were evaporated under reduced pressure and CH_2Cl_2 (20 mL) was added. To this solution was added (*R*)-(+)-glycidol (5.84 mL, 87.9 mmol) and DIPEA (1.53 mL, 8.79 mmol). The reaction was stirred at 40 °C for 16 h. The reaction was washed with H_2O (3 × 20 mL) and brine (3 × 20 mL) and the organic layer was dried over $MgSO_4$. The solvent was removed *in vacuo* and the product was purified using silica column chromatography (EtOAc:n-hexane 1:1 – 4:1) to afford a viscous, colourless oil (2.39 g, 58%).

TLC (EtOAc:n-hexane 4:1) R_f = 0.36; ^{1}H NMR (400 MHz, $(CD_3)_2CO$) δ 7.86 (d, *J* = 7.4 Hz, 2H, Fmoc-Ar), 7.73 (d, *J* = 7.4 Hz, 2H, Fmoc-Ar), 7.42 (t, *J* = 7.4 Hz, 2H, Fmoc-Ar), 7.33 (t, *J* = 7.4 Hz, 2H, Fmoc-Ar), 6.87 (d, *J* = 8.3 Hz, 1H, NH), 4.42 – 4.30 (m, 3H, Fmoc-CH_2, Cys-αCH), 4.30 – 4.21 (m, 1H, Fmoc-CH), 4.00 (d, *J* = 4.4 Hz, 1H, CH-O$\underline{H}$), 3.84 –3.76 (m, 1H, *S*-glyceryl-CH), 3.74 – 3.66 (m, 1H, CH_2-O$\underline{H}$), 3.62 – 3.51 (m, 2H, *S*-glyceryl-*O*CH_2), 3.08 (dd, *J* = 13.3, 5.0 Hz, 1H, Cys-βC$\underline{H}_a H_b$), 2.97 (dd, *J* = 13.3, 5.0 Hz, 1H, Cys- βC$H_a\underline{H}_b$), 2.87 – 2.82 (m, 1H, *S*-glyceryl- C$\underline{H}_a H_b$) 2.66 (dd, *J* = 13.7, 7.9 Hz, 1H, *S*-glyceryl-C$H_a\underline{H}_b$), 1.47 (s, 9H, *t*Bu-CH_3) ppm; ^{13}C NMR (101 MHz, $(CD_3)_2CO$) δ 170.1 (Cys C=O), 156.6 (Fmoc-NH), 144.2 (Fmoc-qC), 141.2 (Fmoc-qC), 127.7 (Fmoc-Ar-CH), 127.1 (Fmoc-Ar-CH), 125.3 (Fmoc-Ar-CH), 119.9 (Fmoc-Ar-CH), 81.3 (*t*Bu-qC), 71.8 (*S*-glyceryl-CH), 66.5 (Fmoc-CH_2), 64.9 (*S*-glyceryl-*O*CH_2), 55.1 (Cys-αCH), 47.1 (Fmoc-CH), 36.0 (*S*-glyceryl-CH_2), 34.6 (Cys-βCH_2), 27.3 (*t*Bu-CH_3) ppm; *m/z* HRMS ($APCI^-$) calculated. $C_{25}H_{31}ClNO_6S$ = 508.1566 $(M + Cl)^-$. Found = 508.1551.

(*R*)-3-(((*R*)-2-((((9*H*-Fluoren-9-yl)methoxy)carbonyl)amino)-3-(*tert*-butoxy)-3-oxopropyl)thio)propane-1,2-diyl dipalmitate **(4)**

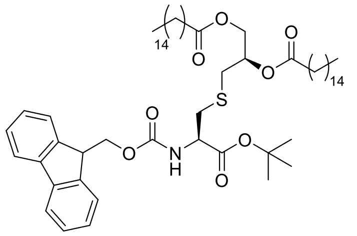

To a solution of palmitic acid (0.88 g, 3.42 mmol) in anhydrous CH_2Cl_2 (50 mL) under argon at 0 °C was added EDC·HCl (0.66 g, 3.42 mmol) and DMAP (0.033 g, 0.27 mmol). The reaction was stirred at 0 °C for 1 h. Diol **3** (0.69 g, 1.37 mmol) was dissolved in anhydrous CH_2Cl_2 (10 mL) and added to the reaction which was stirred at rt under argon for 16 h. The solvent was removed *in vacuo* and the product was purified using silica column chromatography (n-hexane - EtOAc:n-hexane 1:9) to yield a white solid (1.22 g, 94%).

TLC (EtOAc:n-hexane 1:4) R_f = 0.60; ^{1}H NMR (400 MHz, $CDCl_3$) δ 7.76 (d, *J* = 7.4 Hz, 2H, Fmoc-Ar), 7.62 (d, *J* = 7.4 Hz, 2H, Fmoc-Ar), 7.40 (t, *J* = 7.4 Hz, 2H, Fmoc-Ar), 7.31 (t, *J* = 7.4 Hz, 2H, Fmoc-Ar), 5.69 (d, *J* = 7.6 Hz, 1H, NH), 5.20 - 5.12 (m, 1H, *S*-glyceryl-CH), 4.54 – 4.47 (m, 1H, Cys-αCH), 4.44 – 4.30 (m, 3H, Fmoc-CH_2, *S*-glyceryl-*O*C$\underline{H}_a H_b$), 4.24 (t, *J* = 7.1 Hz, 1H, Fmoc-CH), 4.16 (dd, *J* = 11.8, 5.9 Hz, 1H, *S*-glyceryl-*O*C$H_a\underline{H}_b$), 3.11 (dd, *J* = 13.7, 4.9 Hz, 1H, Cys-βC$\underline{H}_a H_b$), 3.04 (dd, *J* = 13.7, 4.9 Hz, 1H, Cys-βC$H_a\underline{H}_b$), 2.77 (d, *J* = 6.3 Hz, 2H, *S*-glyceryl-CH_2), 2.33 – 2.26 (m, 4H, Pal-αCH_2 x2), 1.69 – 1.54 (m, 4H, Pal-CH_2 x2), 1.49 (s, 9H, *t*Bu-CH_3), 1.35 – 1.20 (m, 48H, Pal-CH_2), 0.88 (t, *J* = 6.8 Hz, 6H, Pal-CH_3 x2) ppm; ^{13}C NMR (101 MHz, $CDCl_3$) δ 173.5 (Pal C=O), 173.2 (Pal C=O), 169.6 (Cys C=O), 155.9 (Fmoc C=O), 144.0 (Fmoc-qC), 141.4 (Fmoc-qC), 127.9 (Fmoc-Ar-CH), 127.2 (Fmoc-Ar-CH), 125.3 (Fmoc-Ar-CH), 120.1 (Fmoc-Ar-CH), 83.2 (*t*Bu-qC), 70.4 (*S*-glycery-CH), 67.4 (Fmoc-CH_2), 63.6 (*S*-glyceryl-*O*CH_2), 54.5 (Cys-αCH), 47.3 (Fmoc-CH), 35.5 (Cys- βCH_2), 34.4 (Pal-CH_2), 34.2 (Pal-CH_2), 33.5 (*S*-glycery-CH_2), 32.1 (Pal-CH_2), 29.9 (Pal-CH_2), 29.8 (Pal-CH_2), 29.8 (Pal-CH_2), 29.6 (Pal-CH_2), 29.5 (Pal-CH_2), 29.4 (Pal-CH_2), 29.3 (Pal-CH_2), 29.3 (Pal-CH_2), 28.1 (*t*Bu-CH_3), 25.0 (Pal-CH_2), 25.0 (Pal-CH_2), 22.8 (Pal-CH_2), 14.3 (Pal-CH_3) ppm; *m/z* HRMS ($APCI^+$) calculated. $C_{57}H_{92}NO_8S$ = 950.6538 $(M + H)^+$. Found = 950.6556.

N-(((9*H*-fluoren-9-yl)methoxy)carbonyl)-*S*-((*R*)-2,3-bis(palmitoyloxy)propyl)-L-cysteine **(5)**

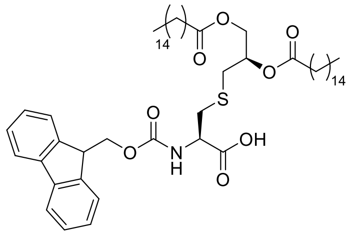

To a solution of compound **4** (1.22 g, 1.28 mmol) in CH_2Cl_2 (3 mL) was added TES (0.5 mL, 3.13 mmol) and TFA (7 mL). The reaction was stirred at room temperature for 16 h. The solvents were removed *in vacuo* to yield the product as a white solid (1.13 g, 99%).

TLC (CH_2Cl_2:MeOH 9:1) R_f = 0.53; ^{1}H NMR (400 MHz, $CDCl_3$) δ 7.76 (d, *J* = 7.4 Hz, 2H, Fmoc-Ar), 7.60 (d, *J* = 7.4 Hz, 2H, Fmoc-Ar), 7.40 (t, *J* = 7.4 Hz, 2H, Fmoc-Ar), 7.31 (t, *J* = 7.4 Hz, 2H, Fmoc-Ar), 5.76 (d, *J* = 7.8 Hz, 1H, NH), 5.21 – 5.13 (m, 1H, *S*-glyceryl-CH), 4.69 – 4.62 (m, 1H, Cys-αCH), 4.41 (d, *J* = 7.0 Hz, 2H, Fmoc-CH_2), 4.35 (dd, *J* = 11.8, 3.4 Hz, 1H, *S*-glyceryl-*O*C$\underline{H}_a H_b$), 4.24 (t, *J* = 7.0 Hz, 1H, Fmoc-CH), 4.15 (dd, *J* = 11.8, 5.9 Hz, 1H, *S*-glyceryl-*O*C$H_a\underline{H}_b$), 3.16 (dd, *J* = 13.4, 4.9 Hz, 1H, Cys-βC$\underline{H}_a H_b$), 3.08 (dd, *J* = 13.4, 4.9 Hz, 1H, Cys-βC$H_a\underline{H}_b$), 2.83 – 2.69 (m, 2H, *S*-glyceryl-CH_2), 2.34 – 2.27 (m, 4H, Pal-αCH_2 x2), 1.69 – 1.54 (m, 4H, Pal-CH_2 x2), 1.34 – 1.21 (m, 48H, Pal-CH_2), 0.88 (t, *J* = 6.8 Hz, 6H, Pal-CH_3 x2) ppm; ^{13}C NMR (101 MHz, $CDCl_3$) δ 174.2 (Pal C=O), 174.1 (Pal C=O), 174.0 (Cys C=O), 156.42 (Fmoc-C=O), 143.7 (Fmoc-qC), 141.47 (Fmoc-qC), 128.0 (Fmoc-Ar-CH), 127.3 (Fmoc-Ar-CH), 125.2 (Fmoc-Ar-CH), 120.2 (Fmoc-Ar-CH), 70.5 (*S*-glycery-CH), 67.8 (Fmoc-CH_2), 63.8 (*S*-glyceryl-*O*CH_2), 53.7 (Cys-αCH), 47.2 (Fmoc-CH), 34.5 (Cys-βCH_2), 34.3 (Pal-CH_2), 33.1 (*S*-glycery-CH_2), 32.1 (Pal-CH_2), 29.9 (Pal-CH_2), 29.8 (Pal-CH_2), 29.6 (Pal-CH_2), 29.5 (Pal-CH_2), 29.4 (Pal-CH_2), 29.3 (Pal-CH_2), 29.2 (Pal-CH_2), 25.0 (Pal-CH_2), 25.0 (Pal-CH_2), 22.8 (Pal-CH_2), 14.3 ((Pal-CH_3) ppm; *m/z* HRMS (APCI$^+$) calculated. $C_{53}H_{84}NO_8S$ = 894.5912 $(M + H)^+$. Found = 894.5927.

(*S*)-2-((((9*H*-fluoren-9-yl)methoxy)carbonyl)amino)-3-(4-hydroxy-3-nitrophenyl)propanoic acid – Fmoc-Tyr(3-NO_2)-OH **(6)**

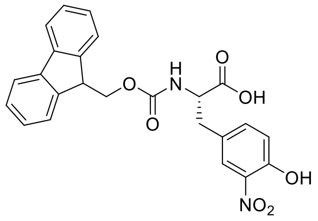

The compound was synthesized according to a known literature procedure. Spectral data were in good agreement with the literature[12]. To a suspension of 3-nitrotyrosine (2.00 g, 8.85 mmol) in H_2O (150 mL) at 0 °C was added a solution of Fmoc-*O*Su (2.99 g, 8.85 mmol) in acetone (100 mL). The pH of the reaction was adjusted to pH 9 using aq. $NaHCO_3$ solution (10 %(w/v). The reaction was stirred at room temperature for 16 h resulting in the formation of a clear yellow solution. The acetone was removed under reduced pressure and the resulting aqueous solution was washed with Et_2O (3 × 100 mL). The aqueous layer was acidified to pH 3 resulting in the precipitation of a yellow solid. This was extracted with EtOAc (5 × 50 mL) and the combined organic layers were washed with water (3 × 100 mL) and brine (3 × 100 mL), dried over $MgSO_4$ and filtered. The solvents were removed *in vacuo* yielding the product as a yellow powder (2.96 g, 77%).

TLC (CH_2Cl_2:MeOH 9:1) R_f = 0.22; ^{1}H NMR (400 MHz, $CDCl_3$) δ 10.48 (s, 1H, Phenol-OH), 7.91 (s, 1H, Tyr-Ar), 7.76 (d, *J* = 7.5 Hz, 2H, Fmoc-Ar), 7.54 (t, *J* = 7.5 Hz, 2H, Fmoc-Ar), 7.39 (t, *J* = 7.5 Hz, 2H, Fmoc-Ar), 7.34 – 7.27 (m, 3H, Fmoc-Ar x 2, Tyr-Ar), 7.06 (d, *J* = 8.4 Hz, 1H, Tyr-Ar), 5.25 (d, *J* = 7.7 Hz, 1H, NH), 4.70 – 4.64 (m, 1H, Tyr-αCH), 4.54 – 4.35 (m, 2H, Fmoc-CH_2), 4.19 (t, *J* = 6.4 Hz,

1H, Fmoc-CH), 3.25 – 3.00 (m, 2H, Tyr-βCH_2) ppm; ^{13}C NMR (101 MHz, $CDCl_3$) δ 173.8 (COOH), 155.8 (Fmoc C=O),154.4 (Tyr-qC-OH), 143.7 (Fmoc-qC), 141.5 (Fmoc-qC), 138.9 (Tyr-Ar-CH), 133.5 (Tyr-qC), 128.0 (Fmoc-Ar-CH), 127.3 (Fmoc-Ar-CH), 125.4 (Tyr-Ar-CH), 125.1 (Fmoc-Ar-CH), 120.5 (Tyr-Ar-CH), 120.2, 120.2 (Fmoc-Ar-CH), 67.2 (Fmoc-CH_2), 54.4 (Tyr- αCH), 47.3 (Fmoc-CH), 36.9 (Tyr-βCH_2) ppm; *m/z* HRMS (ESI^+) calculated. $C_{24}H_{20}N_2NaO_7$ = 471.1163 $(M + Na)^+$. Found = 471.1167.

2-((*tert*-Butoxycarbonyl)amino)benzoic acid – Boc-Abz-OH **(7)**

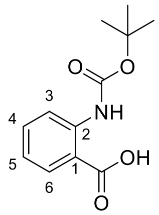

The compound was synthesized according to a known literature procedure. Spectral data were in good agreement with the literature[13]. To a solution of 2-aminobenzoic acid (5.00 g, 36.5 mmol) in THF:H_2O (1:1, 80 mL) was added aq. NaOH solution (2 M) until pH 10 was reached. Boc_2O (8.75 g, 40.1 mmol) was added and the reaction was stirred at room temperature for 16 h. THF was evaporated under reduced pressure and the aqueous layer acidified to pH 4 using aqueous citric acid solution (15 %(w/v) resulting in the formation of a white precipitate. The precipitate was filtered and washed with H_2O (3 × 50 mL). The solid was dissolved in EtOAc (100 mL) and washed with H_2O (3 × 100 mL) and brine (100 mL). The combined organic layers were washed with brine, dried over $MgSO_4$ and filtered. The solvent removed *in vacuo* yielding the compound as a white solid. (6.41 g, 74%).

TLC (CH_2Cl_2:MeOH 9:1) R_f = 0.56; ^1H NMR (400 MHz, $CDCl_3$) δ 10.00 (s, 1H, NH), 8.45 (d, *J* = 8.0 Hz, 1H, Ar-H3), 8.10 (dd, *J* = 8.0 Hz, *J* = 1.5 Hz, 1H, Ar-H6), 7.55 (dt, *J* = 8.0 Hz, *J* = 1.5 Hz, 1H, Ar-H4), 7.06 – 6.97 (m, 1H, Ar-H5), 1.54 (s, 9H, Boc-CH_3) ppm; ^{13}C NMR (101 MHz, $CDCl_3$) δ 173.1 (COOH), 152.7 (Boc C=O), 142.9 (Ar-C1), 135.6 Ar-C4), 131.9 (Ar-C6), 121.3 (Ar-C5), 119.0 (Ar-C3), 113.3 (Ar-C2), 80.9 (Boc-qC), 28.3 (Boc-CH_3) ppm; *m/z* HRMS (ESI^+) calculated. $C_{12}H_{15}NNaO_4$ = 260.0893 $(M + Na)^+$. Found = 260.0898.

NMR spectra of peptide 1 are shown in Supplementary Figs. 23 – 26.

Supplementary Figures

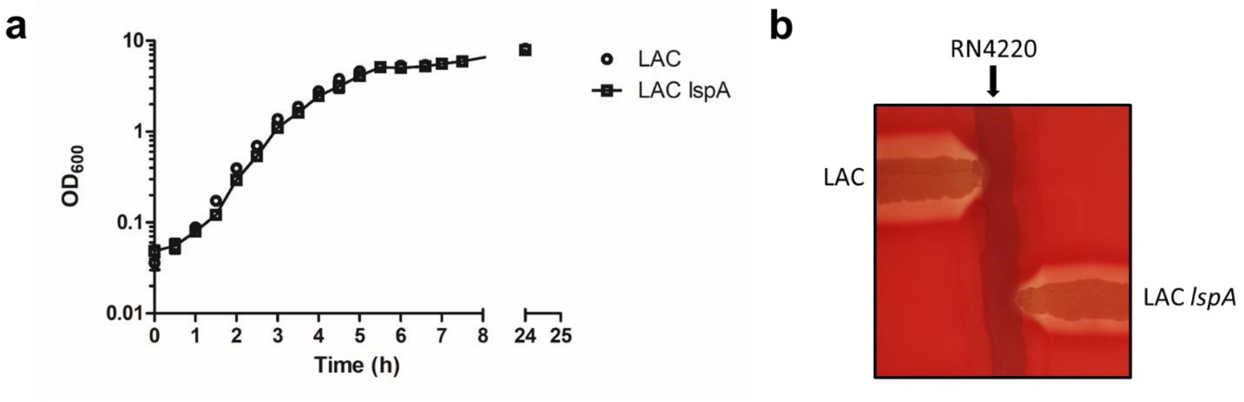

Supplementary Figure 1. Wild-type and mutant *S. aureus* strains were phenotypically indistinguishable in terms of growth profile in tryptic soy broth and haemolysis patterns on sheep blood agar. (a) Growth curves for *S. aureus* LAC (wild-type) and LAC lspA (mutant) in tryptic soy broth show data from two independent experiments. Mean values ± SD are plotted. **(b)** Haemolytic profiles of *S. aureus* LAC and LAC *lspA* cross-streaked perpendicularly to *S. aureus* RN4220[14] on sheep blood agar. Plates were incubated at 37 °C for 24h. Source data are provided as a Source Data file.

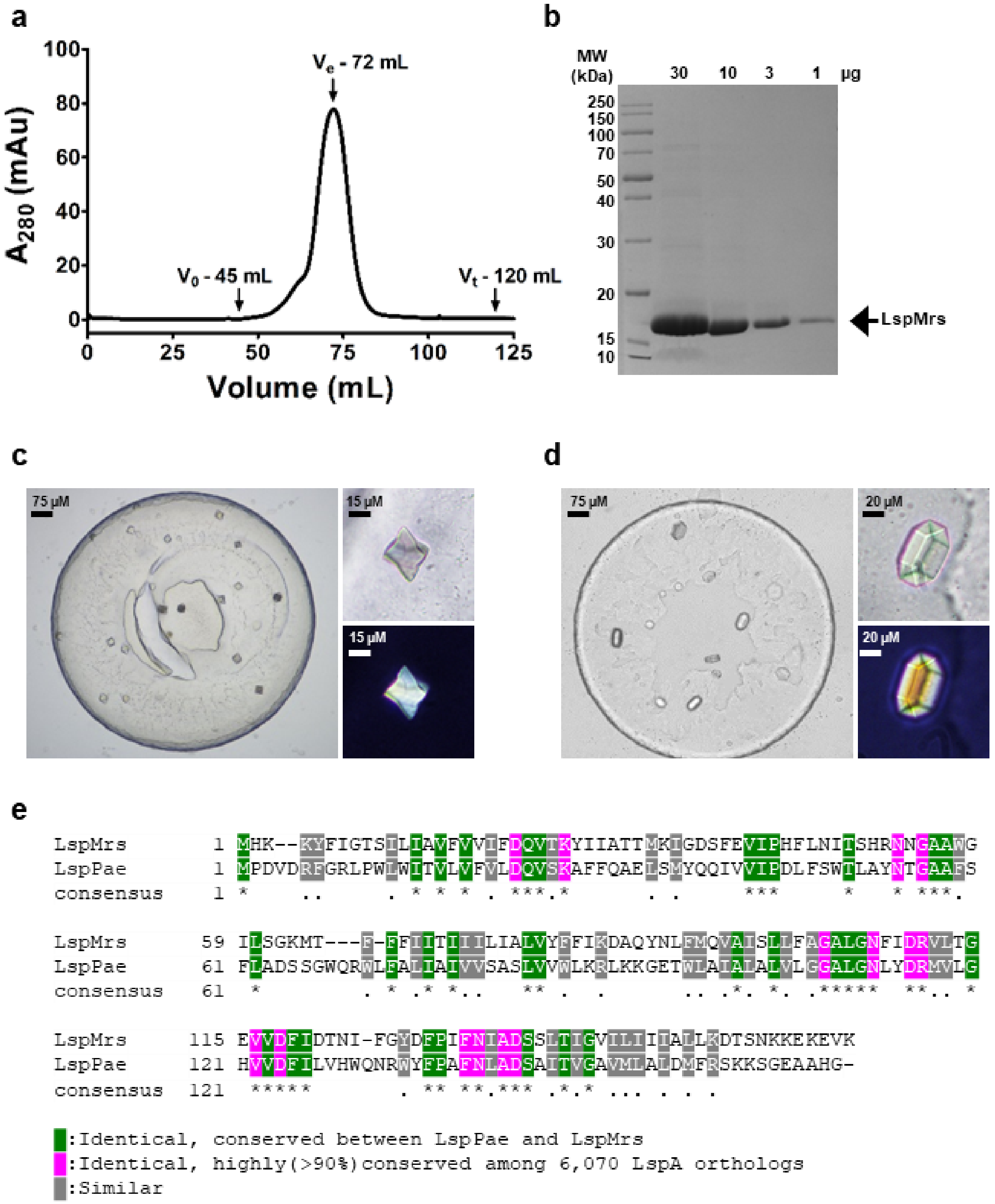

Supplementary Figure 2. Properties of the LspMrs ortholog used in this study. (**a**) Size-exclusion chromatogram. (**b**) Loading series analysis by SDS-PAGE shows that the protein is >95% pure. LspPae was of equally good quality[1]. Molecular weight standards are in the left lane. (**c**) Crystals of the LspMrs-globomycin complex. Left panel: LCP bolus. Right panels: enlarged view of a typical LspMrs-globomycin crystal viewed with brightfield light (top) and between cross polarizers (bottom). (**d**) Crystals of the LspMrs-myxovirescin complex. Left panel: LCP bolus. Right panels: enlarged view of a typical LspMrs-myxovirescin crystal viewed with brightfield light (top) and between cross polarizers (bottom). (**e**) Sequence alignment of LspMrs (Uniprot ID: Q6GHN9) and LspPae (Uniprot ID: Q9HVM5). The fourteen highly conserved residues in and around the active and binding sites of LspA are highlighted in magenta. Sequence identity is 31% and sequence similarity is 52%.

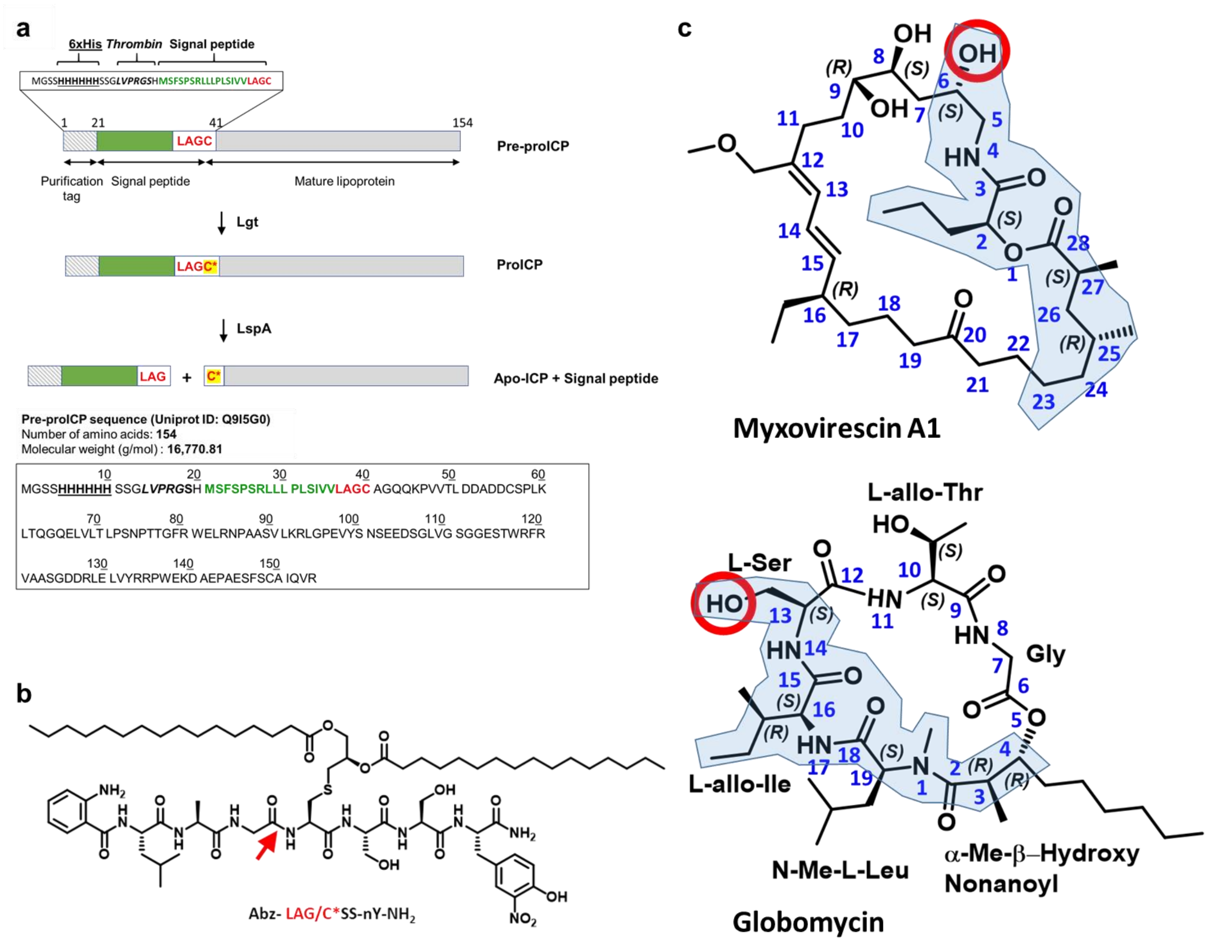

Supplementary Figure 3. LspA substrates and antibiotics used in this study. (**a**) Cartoon representation of the ICP substrates and products in the lipoprotein processing pathway involving the Lgt and LspA enzymes. The amino acid sequence of the full-length pre-prolipoprotein is included. (**b**) FRET lipopeptide. Abz corresponds to the fluorophore, aminobenzoic acid, C* to the dagylated lipobox cysteine, and nY-NH2 to the amide form of the quencher, nitro-tyrosine. The scissile bond is marked with a red arrow. (**c**) Myxovirescin and globomycin with ring atoms numbered in blue and blocking hydroxyls marked with a red circle. Atoms and groups that overlap in the superpose of the LspMrs-myxovirescin and LspMrs-globomycin complex structures (Fig. 3), the spine atoms, are shaded light blue. The propyl group on ring atom 2 in myxovirescin and the butyl group on ring atom 16 in globomycin overlap in the superpose and are referred to in the text.

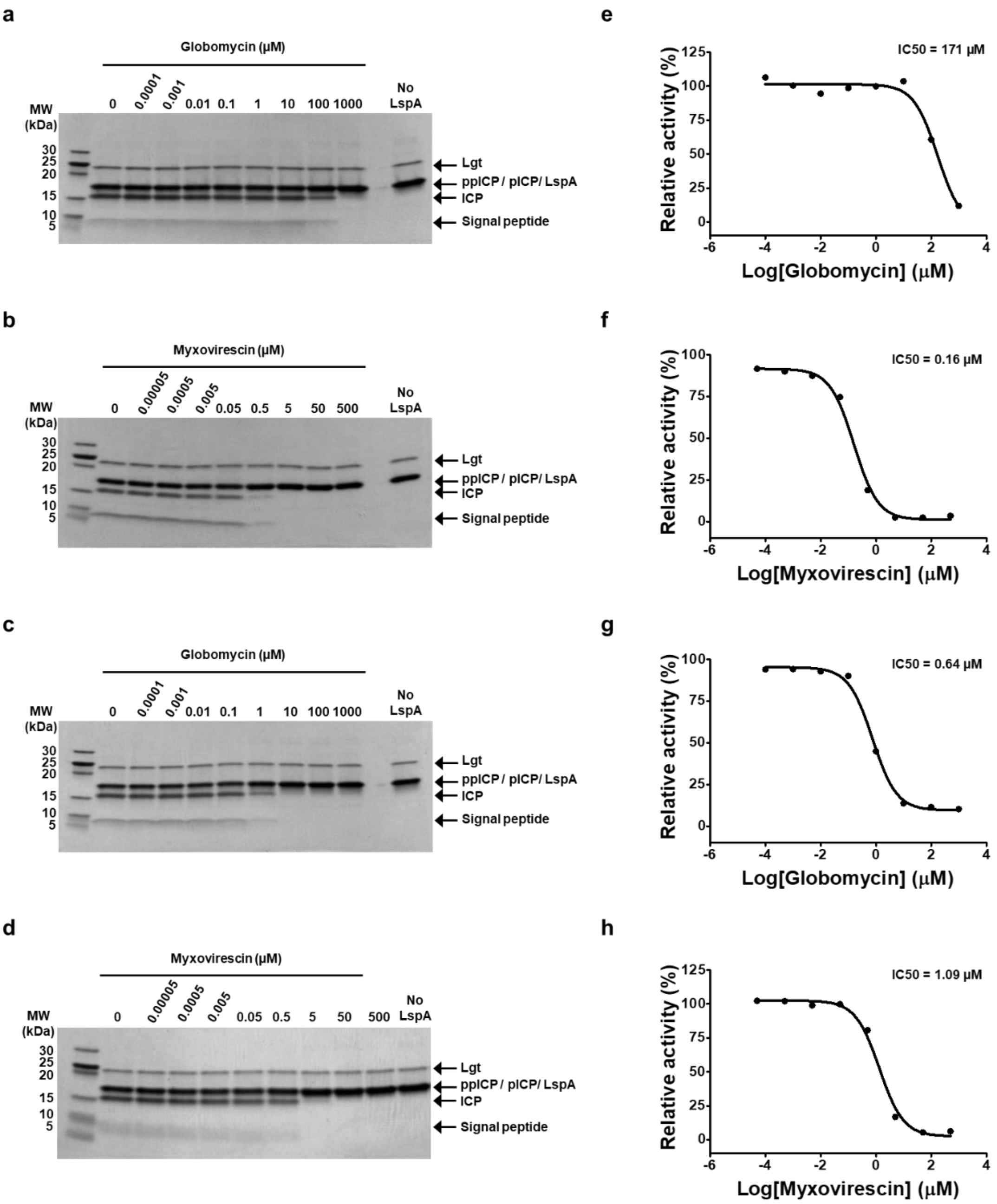

Supplementary Figure 4. Peptidase activity of LspMrs and LspPae with a prolipoprotein substrate in the presence and absence of globomycin and myxovirescin. (**a-d**) Gel-shift assays performed using the prolipoprotein, proICP, generated *in situ* by the action of Lgt on pre-proICP. (**e-h**) IC_{50} determination for inhibition by globomycin (a, c, e, g) and myxovirescin (b, d, f, h) based on gel-shift data (a-d). Activity was quantified by tracking ICP production. a, b, e, f refer to data for LspMrs. c, d, g, h refer to data for LspPae. The far right lane in each of the four gels has no added LspA and reveals a band for Lgt and a second band which includes co-migrating LspA, pre-proICP and proICP. Molecular weight markers are in the far left lane. All other lanes represent the full assay complement with and without antibiotics. Details are provided in the Methods section. The indicated IC_{50} values are the average of two independent replicate measurements. Source data are provided as a Source Data file.

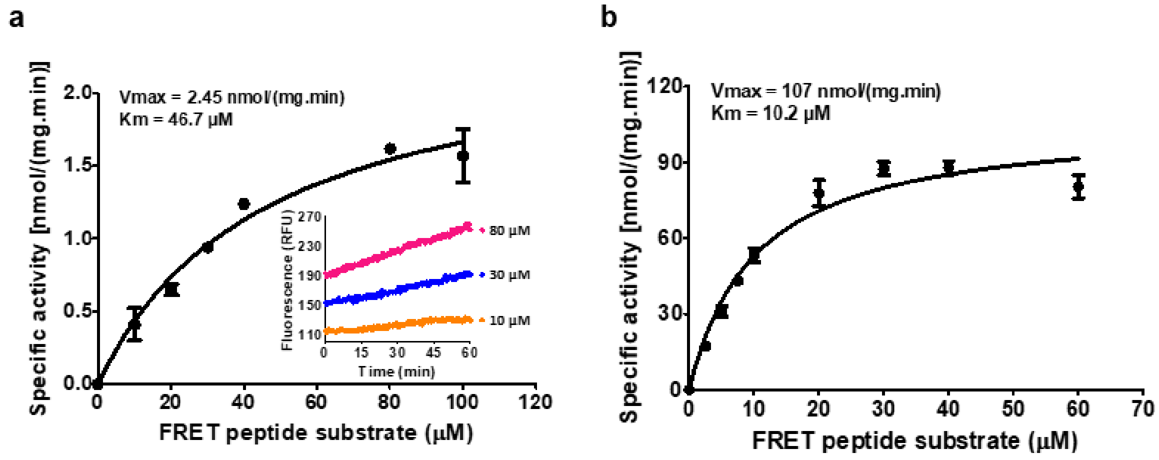

Supplementary Figure 5. Substrate saturation analysis of LspA as monitored by FRET-based assay. (**a**) Substrate saturation plot for LspMrs at an enzyme concentration of 0.3 µM. The inset shows FRET reaction progress curves at three substrate concentrations. (**b**) Substrate saturation plot for LspPae at an enzyme concentration of 0.1 µM. The fitted line in (a) and (b) is based on a simple Michaelis-Menten kinetics model with R^2 values of 0.974 and 0.963 for LspMrs and LspPae, respectively. A model that includes substrate inhibition has a slightly better fit with R^2 values of 0.986 and 0.996, respectively. Given the issues associated with solubilities, discussed under 'Complex nature of the LspA assay reaction mix' (Supplementary Discussion), we only show here the fits with the simpler Michaelis-Menten kinetics model. The data shown correspond to the average ± standard deviation of duplicate independent measurements. Source data are provided as a Source Data file.

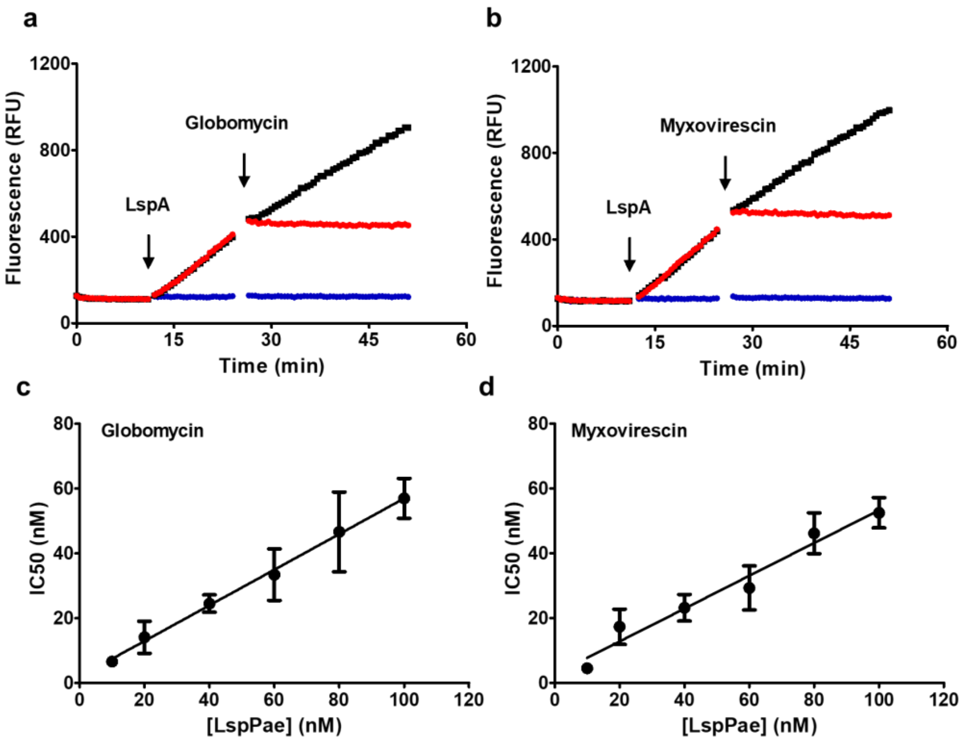

Supplementary Figure 6. LspPae inhibition by globomycin and myxovirescin and effect of enzyme concentration on the IC_{50} values. Evidence for tight binding inhibition. (**a**, **b**) FRET assay progress curves for LspPae as affected by the addition of globomycin and myxovirescin during the course of the peptidase reaction. Each figure has three progress curves: i) uninhibited (black data points), ii) with antibiotic added 20 to 30 min after the start of the reaction (red data points), and iii) an enzyme-free control (blue data points). A 5-fold molar excess of both antibiotics inhibit the enzyme within minutes, at most, indicating that the inhibitor on-rates are fast. There is a 2 to 3 minute gap in the data at around the time the antibiotics are added. The gap arises from the need to stop the fluorescence plate reader in order to add and to mix in antibiotic and to restart the instrument. (**c**) Dependence of IC_{50} values for globomycin on enzyme concentration. (**d**) Dependence of IC_{50} values for myxovirescin on enzyme concentration. Dose-response curves used for IC_{50} determination were performed over a range of antibiotic concentrations up to 500 nM. Error bars represent the 95% confidence intervals in IC_{50} determination on the bases of analysis using Graphpad Prism. The data are consistent with tight inhibitor binding which is described by the relationship: $IC_{50} = K_i' + [E]/2$, where Ki' is the apparent inhibition constant and [E] is total enzyme concentration. For globomycin and myxovirescin, the estimated K_i' values are 1.96 ± 3.0 nM and 2.69 ± 2.8 nM, respectively. Separate analysis, shown below (Supplementary Fig. 18), yielded estimated Ki' values of 740 ± 410 and 47.6 ± 128 pM for globomycin and myxovirescin, respectively. Source data are provided as a Source Data file.

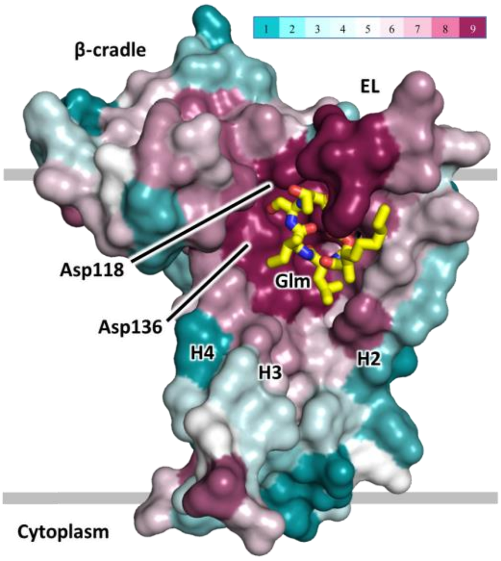

Supplementary Figure 7. Conserved residues cluster around the globomycin binding pocket in LspA from *S. aureus*. Conserved residue surface representation of the protein is based on an analysis of 500 orthologs having 35% to 95% sequence identity with LspMrs (Uniprot ID: Q6GHN9) using the ConSurf server[15]. The view is into the globomycin binding site as in Fig. 3a. Coloring is by conservation value with light blue, white, and purple corresponding to variable, average, and conserved, respectively. Globomycin (Glm) is shown as sticks with yellow carbons. The locations of the catalytic dyad aspartate residues are indicated. Approximate membrane boundary positions are demarked by horizontal lines.

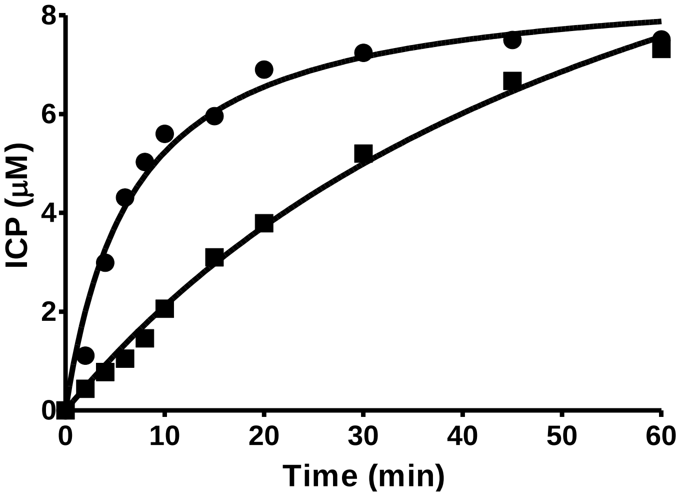

Supplementary Figure 8. Reaction progress curves for LspMrs and LspPae determined by the gel-shift method with proICP as substrate. LspPae (solid circles). LspMrs (solid squares). Progress was quantified by measuring the formation of the ICP product using ImageJ. Assays were performed at 50 mM Tris/HCl pH 7.5, 150 mM NaCl, 1 mM DTT, 0.02 %(w/v) LMNG, 250 µM DOPG, 12 µM pre-proICP, 1.2 µM Lgt and 0.5 µM LspMrs or LspPae. The initial rate (first few minutes of reaction) of peptidolysis by LspPae is estimated at 3-times that of LspMrs. After 30 min, LspPae is 1.33 times more active than LspMrs, as reported in Table 1. Source data are provided as a Source Data file.

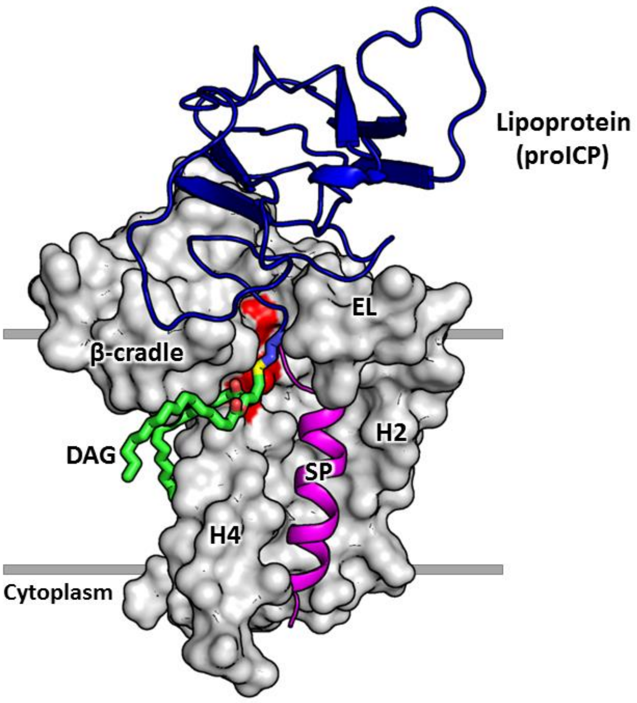

Supplementary Figure 9. Model of the prolipoprotein substrate, proICP, from *P. aeruginosa* docked into the apo form of the LspMrs-globomycin complex structure. The signal peptide (SP, magenta) docks into the pocket created by H2, H3, and H4. The lipobox cysteine, Cys* (sulfur; yellow), is dagylated (green sticks), and the lipoprotein (blue, cartoon) extends across the β-cradle into the extracellular space. Docking details are available in the Methods section. Catalytic aspartates are highlighted in red.

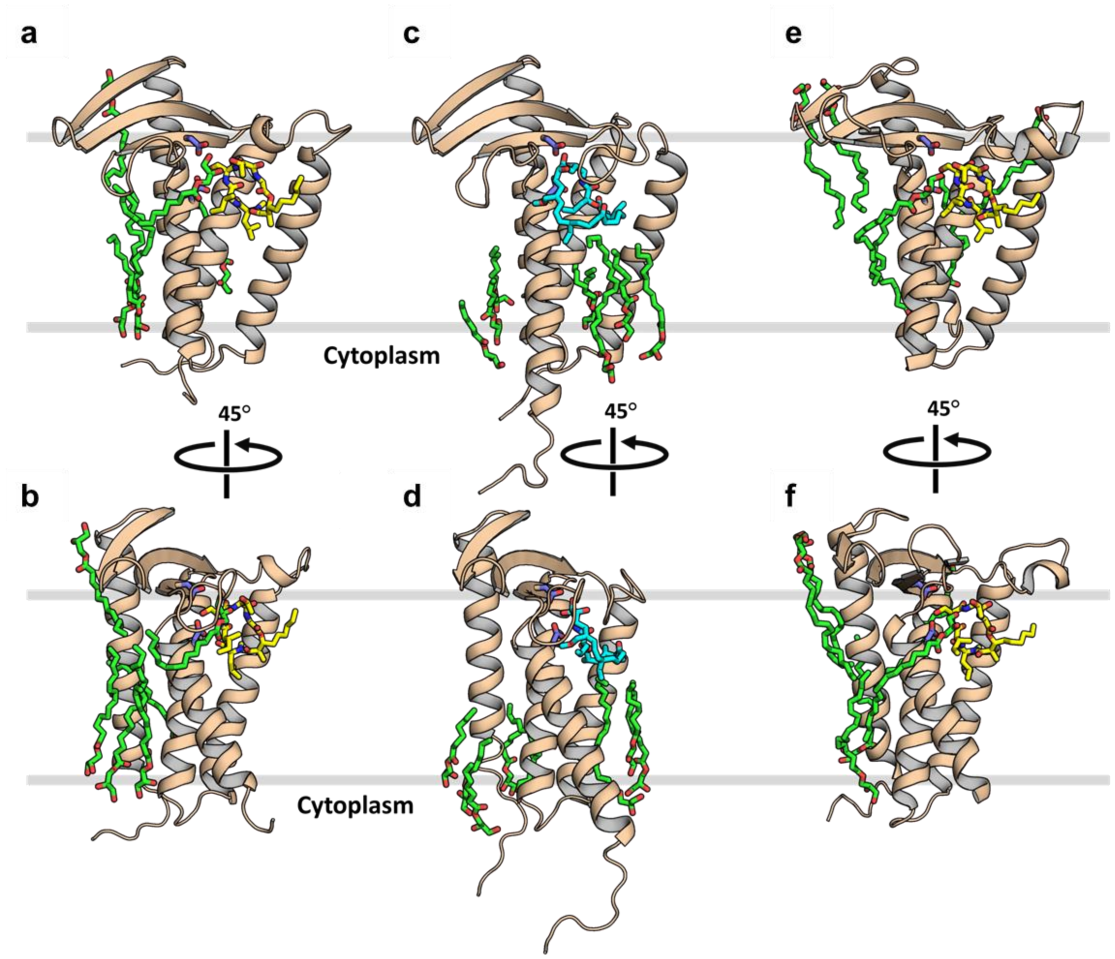

Supplementary Figure 10. Structured monoolein molecules in LspMrs and LspPae complexes with globomycin and myxovirescin. (**a**, **b**) LspMrs-globomycin complex. (**c**, **d**) LspMrs-myxovirescin complex. (**e**, **f**) LspPae-globomycin complex (PDB ID, 5DIR[1]). Monoolein molecules are represented as sticks with green carbons. Globomycin and myxovirescin have yellow and cyan carbons, respectively. Catalytic aspartates have dark blue carbons.

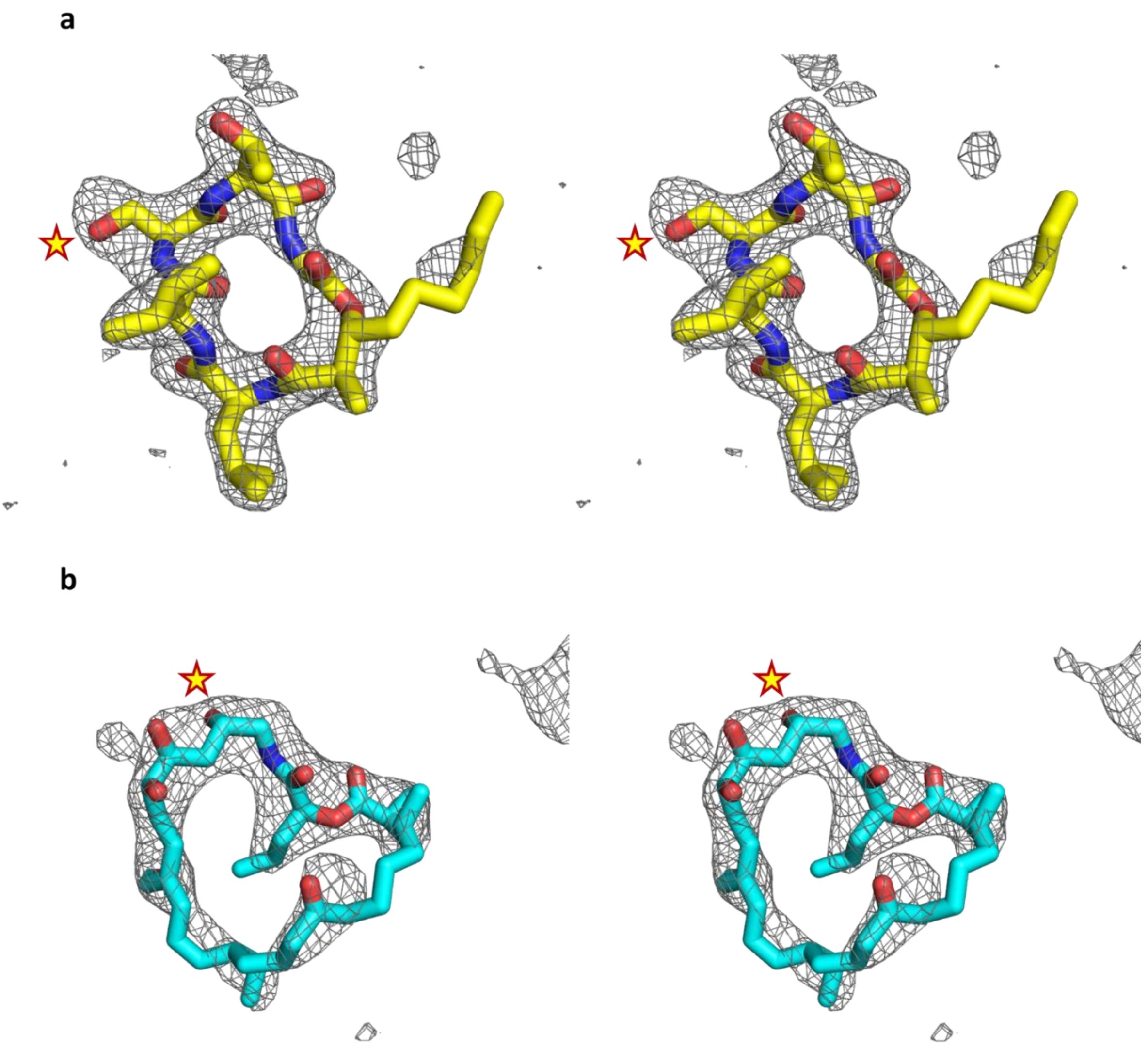

Supplementary Figure 11. Stereo images of Polder-OMIT maps for LspMrs in complex with globomycin and with myxovirescin. (**a**) Complex with globomycin at 1.92 Å resolution. (**b**) Complex with myxovirescin at 2.30 Å resolution. Phenix Polder OMIT map[16] (grey mesh) of the antibiotic globomycin (yellow carbons) and myxovirescin (cyan carbons) are contoured at 3 sigma. A star identifies the blocking hydroxyl that sits between the two catalytic aspartate residues in the active site of LspA.

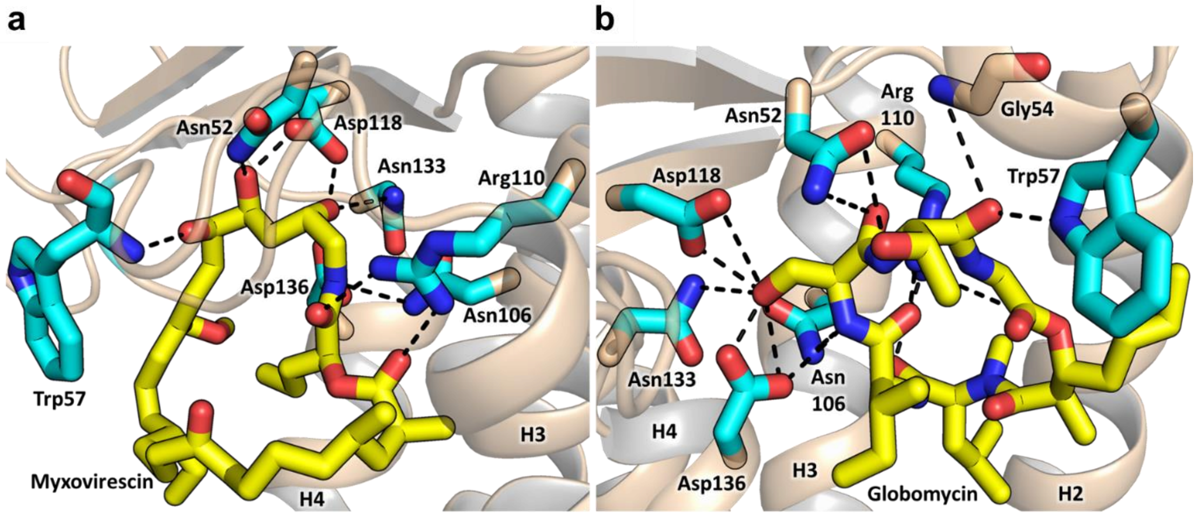

Supplementary Figure 12. Myxovirescin and globomycin interact closely with highly conserved residues in LspMrs. (**a**) Myxovirescin complex. (**b**) Globomycin complex. Antibiotics are shown in the binding pocket with yellow carbons. The views in (a) and (b) are slightly different to best display the interactions. For clarity, some of the protein structure has been rendered partly transparent and not all interacting residues have been included. For reference, all interacting partners and distances are listed in Supplementary Table 4.

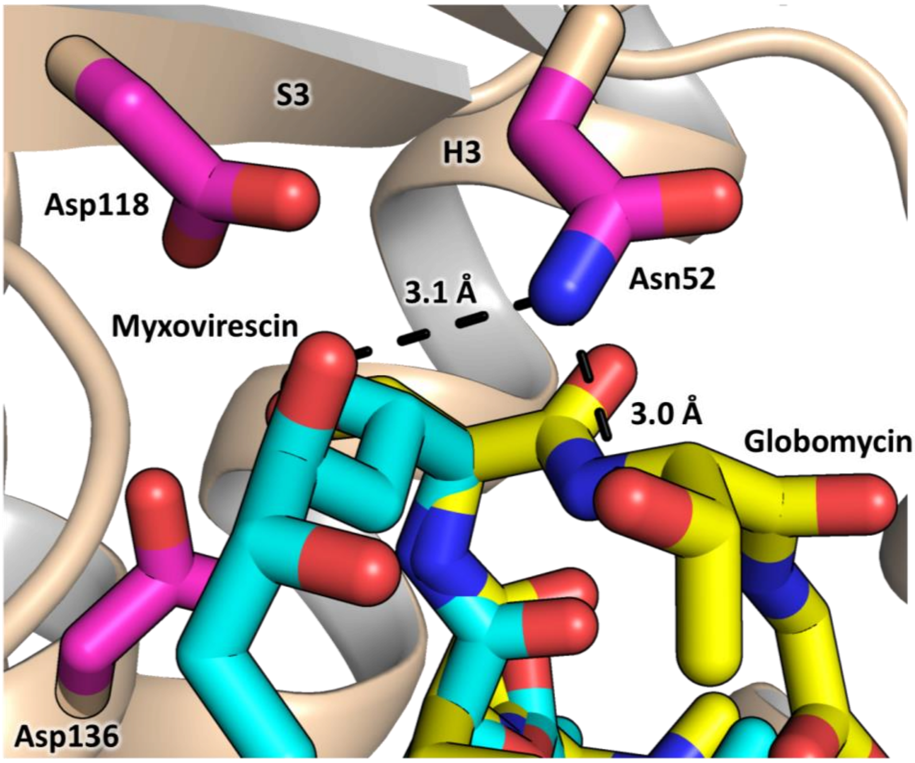

Supplementary Figure 13. Highly conserved residue Asn52 interacts at locations in globomycin and myxovirescin where the two antibiotics diverge in the binding pocket of LspMrs. The crystal structure of the LspMrs-myxovirescin complex is used to show the protein and its critical residues. The LspMrs-globomycin structure and parts of the LspMrs-myxovirescin structure have been omitted for clarity. Catalytic residues Asp118 and Asp136 are included as fiducials. Globomycin and myxovirescin are shown as sticks with yellow and cyan carbon atoms, respectively.

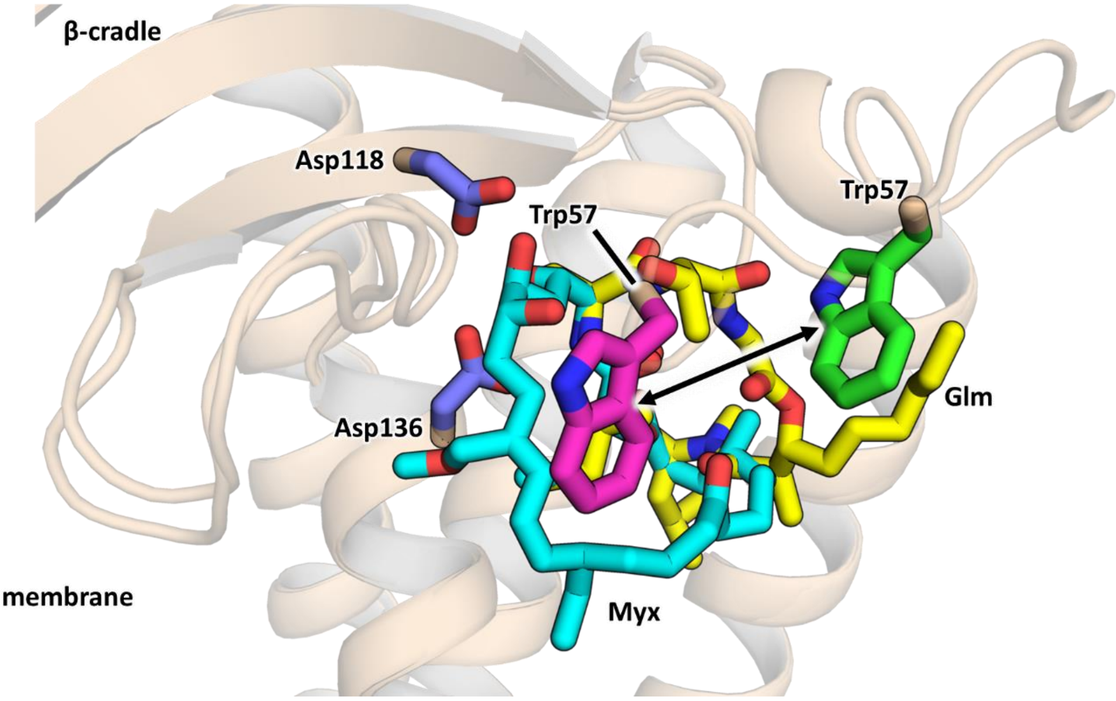

Supplementary Figure 14. Extracellular loop flexibility in LspMrs is apparent in effective myxovirescin and globomycin binding. The centre of mass of Trp57 in the extracellular loop of the enzyme changes location by ~10 Å (double headed arrow) in going from the myxovirescin (magenta) to the globomycin (green) bound form. The view is into the binding pocket of the superposed structures where myxovirescin and globomycin are shown as sticks with cyan and yellow carbon atoms, respectively.

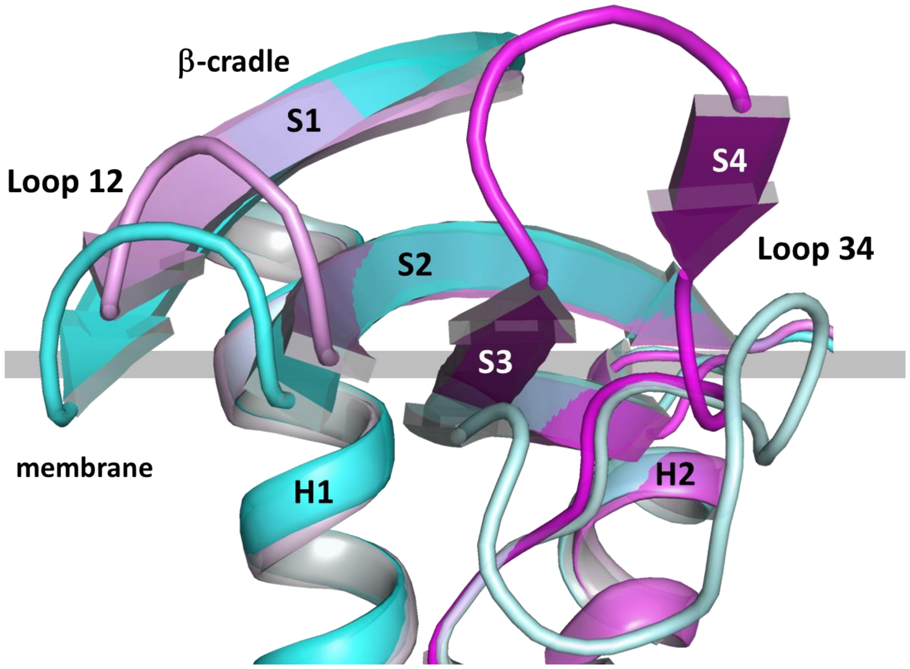

Supplementary Figure 15. Differences in the β-cradle region of the structures of LspMrs and LspPae in complex with globomycin. In LspMrs (cyan), the loop connecting β-strands S3 and S4 faces into the membrane. In LspPae (magenta), it faces away from the membrane.

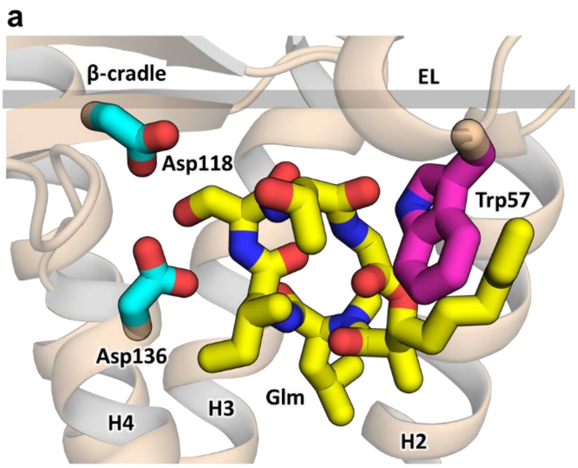

Supplementary Figure 16. Differences in the region of the extracellular loop (EL) of the structures of LspMrs and LspPae in complex with globomycin. (**a**) LspMrs-globomycin complex. (**b**) LspPae-globomycin complex[1]. The EL appears to be flexible with Trp57 and Phe59 serving to clamp globomycin in place in the binding pockets of LspMrs and LspPae, respectively.

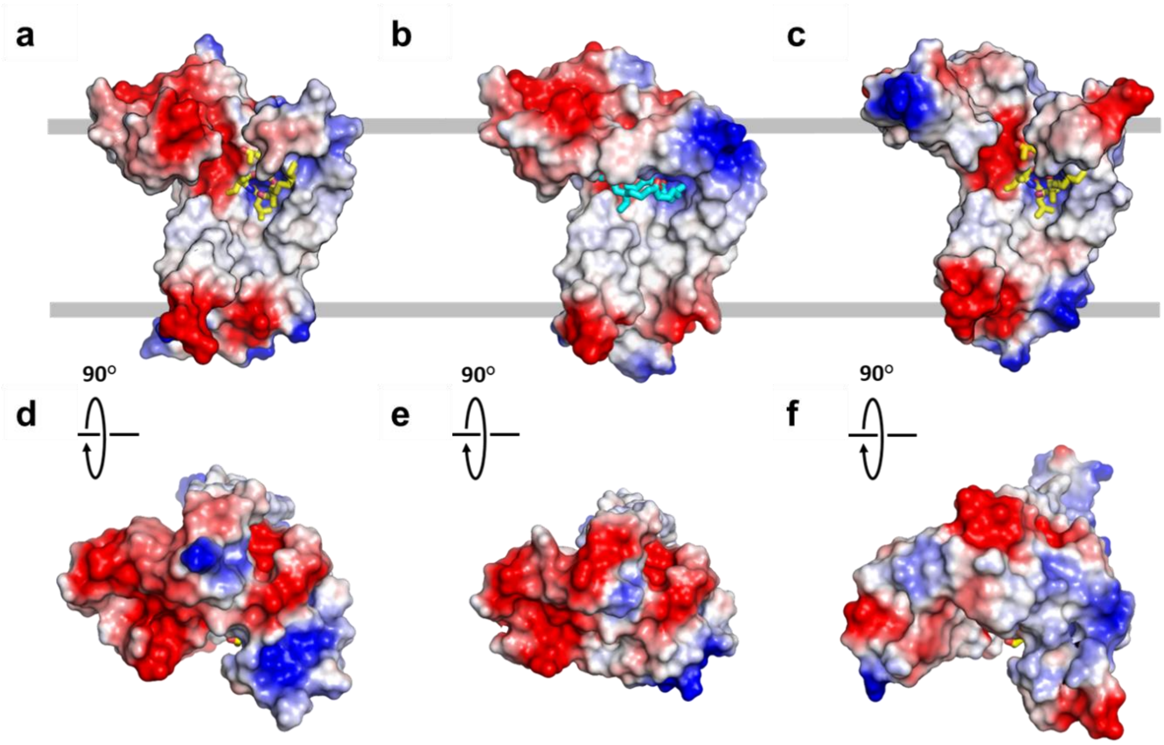

Supplementary Figure 17. Electrostatics of LspMrs and LspPae in complex with globomycin and myxovirescin in surface representation. LspMrs in the left and middle panels. LspPae in the right panel. (**a-c**) View from the membrane with the cytoplasm below the bottom horizontal bar. (**d-f**) View from the extracellular space. Electrostatics range from -5 to 5 KT/e (red, negatively charged; grey/white, neutral; blue, positively charged). Globomycin and myxovirescin are in stick representation with yellow and cyan carbons, respectively. In the myxovirescin complex, the EL completely buries the antibiotic and masks it from access and view from the extracellular space. See (e). In (b), the N-terminal sequence HMHK and C-terminal sequence SNKKEKEVK which extend into the cytoplasm as a helix/coil have been deleted for a more uniform comparison with the other structures (a, c) where these residues are disordered.

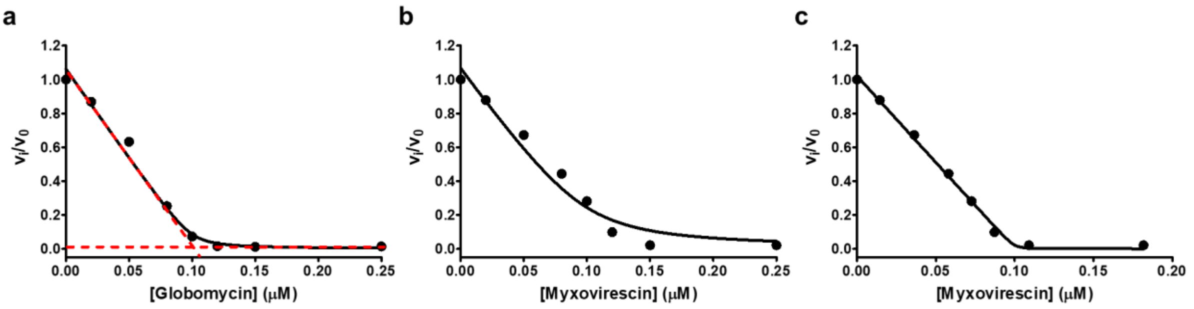

Supplementary Figure 18. Morrison analysis for LspPae active site and myxovirescin concentration determination. (**a**) Effect of globomycin on LspPae reaction rate recorded by FRET-based assay at 30 μM substrate and a nominal, spectroscopically determined LspPae concentration of 0.1 μM. The data are fit with the Morrison equation (Supplementary Equation 1; solid line). Data are shown as the average of duplicate independent measurements. The intersection of the linear extrapolations (dashed lines) from the low and high globomycin concentration portions of the plot at 0.1 μM globomycin corresponds to the concentration of enzyme active sites assuming a single active site per enzyme and that the enzyme functions as a monomer. The two enzyme concentrations agree remarkably well. (**b**, **c**) Effect of myxovirescin on LspPae reaction rate recorded by FRET-based assay at 30 μM substrate and an LspPae concentration of 0.1 μM, as determined in (A). A best fit to the data, using the Morrison equation (Supplementary Equation 1; solid line) assuming the nominal concentration of myxovirescin, is shown in (b). Adjusting the myxovirescin concentration by a factor of 0.73 and rerunning the analysis provides the fit shown in (c). The estimated Ki' values for globomycin and myxovirescin are 740 ± 410 and 47.6 ± 128 pM, respectively. Source data are provided as a Source Data file.

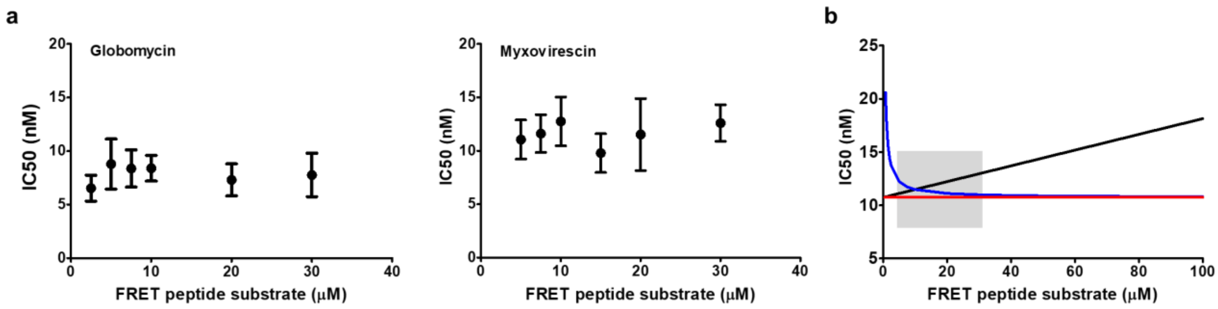

Supplementary Figure 19. Exploring the mechanism by which LspPae is inhibited by globomycin and myxovirescin. (**a**) Experimentally determined dependence of IC_{50} values for globomycin and myxovirescin on substrate concentration at 20 nM LspPae in FRET-based assays. Conditions under which measurements were made include: a substrate concentration range of 2.5 μM (for globomycin) or 5 μM (myxovirescin) to 30 μM and use of an optimized LspPae reaction buffer (100 mM MES/NaOH pH 5.6, 150 mM NaCl and 0.05 %(w/v) LMNG). Assays were performed at 37 ºC. Dose-response curves used for IC_{50} determination were performed over a range of antibiotic concentrations up to 100 nM. The error bars shown are the 95% confidence intervals in IC_{50} determination on the bases of analysis using Graphpad Prism. IC_{50} values for globomycin and myxovirescin show no significant dependence on substrate concentration in the range investigated. The average IC_{50} value for both antibiotics is close to 10 nM corresponding to half the enzyme concentration [E] used for assay. (**b**) Predicted dependence of IC_{50} on substrate concentration based on the relationship for uncompetitive inhibition: $IC_{50} = K_i(1+K_m/[S]) + [E]/2$ (blue line), competitive inhibition: $IC_{50} = K_i(1+[S]/K_m) + [E]/2$ (black line), and for non-competitive inhibition: $IC_{50} = (([S]+K_m)/((K_m/K_i)+([S]/\alpha K_i))) + [E]/2$ (red line). When $\alpha = 1$, simple non-competitive inhibition applies. Mixed inhibition applies when $\alpha \neq 1$ (Supplementary Fig. 20). The shaded region corresponds to the limits on the error bars for myxovirescin in (a). Clearly, the error in the experimental IC_{50} data is too large to enable a determination of inhibition type. Source data are provided as a Source Data file.

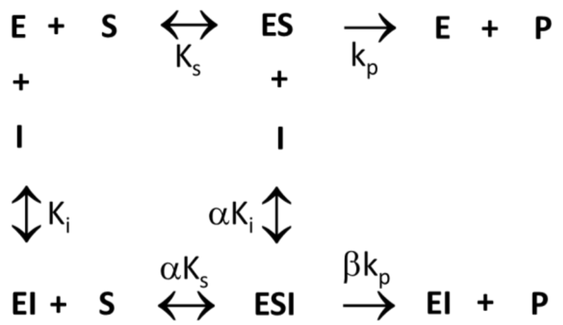

Supplementary Figure 20. Reaction scheme for an enzyme combining reversibly with a substrate and an inhibitor. In competitive inhibition, the inhibitor interacts with the free enzyme, E. In uncompetitive inhibition, it interacts with the enzyme-substrate complex, ES. Under non-competitive inhibition conditions, the inhibitor can react with the free enzyme and the enzyme-substrate complex, and the substrate can bind with the enzyme-inhibitor complex, EI. Mixed inhibition is observed when α is >1 or is <1. It is assumed that in most situations, the ternary ESI complex does not yield product, in which case $\beta = 0$. Partial inhibition occurs in those special cases where $\beta > 0$. K_s and K_i are the substrate binding and inhibition constants, respectively. k_p is the catalytic rate constant.

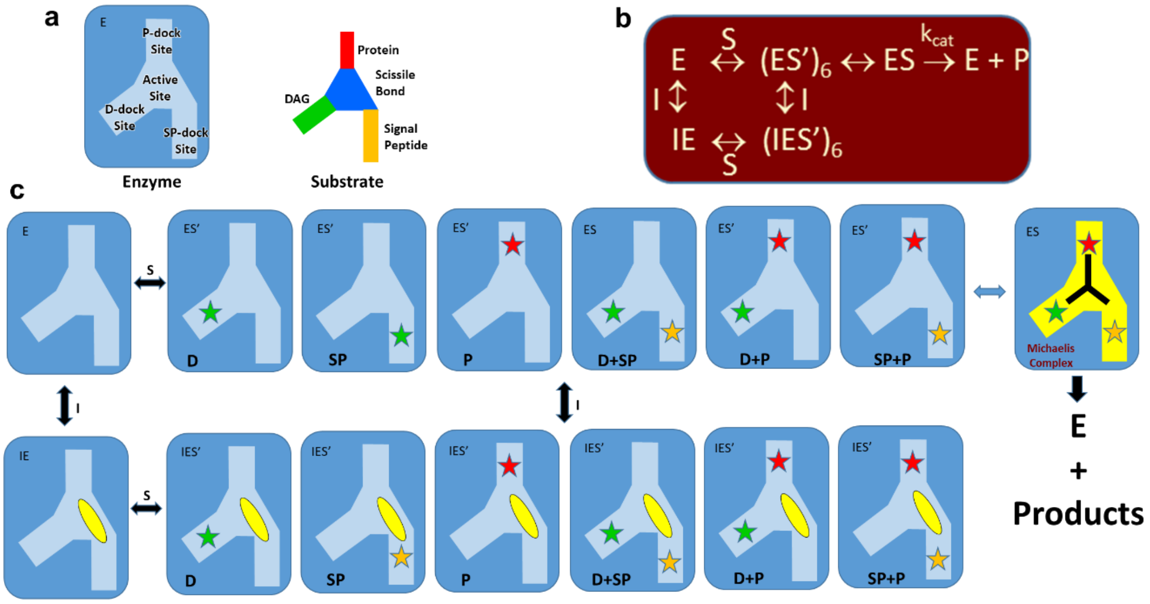

Supplementary Figure 21. Multiple ways by which LspA might interact with its lipoprotein substrate and an inhibitor such as globomycin or myxovirescin. (**a**) Complementary features of the enzyme and the substrate based on crystal structures of LspA and on the proposed ES complex model in Supplementary Fig. 9. (**b**) Possible modes by which a substrate and an orthosteric inhibitor can interact with an enzyme. (**c**) Cartoon representation of how a lipoprotein substrate with flexible parts and a relatively rigid inhibitor molecule like globomycin and myxovirescin might interact with LspA with its proposed four docking sites. The green, red and orange stars represent, respectively, the DAG (D), protein (P) and signal peptide (SP) parts of the substrate. The yellow oval represents the inhibitor.

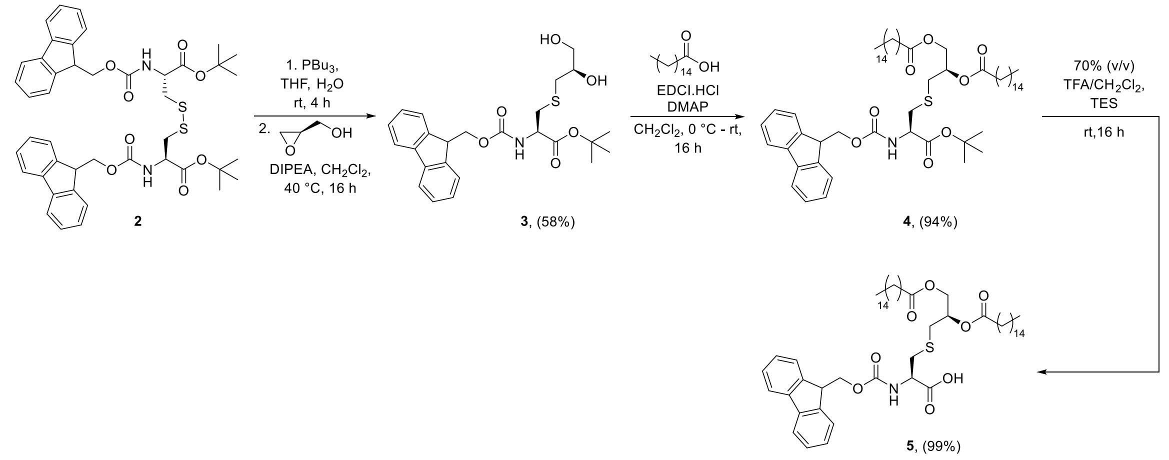

Supplementary Figure 22. Synthetic scheme of Fmoc-Cys(Pam)$_2$-OH (**5**)

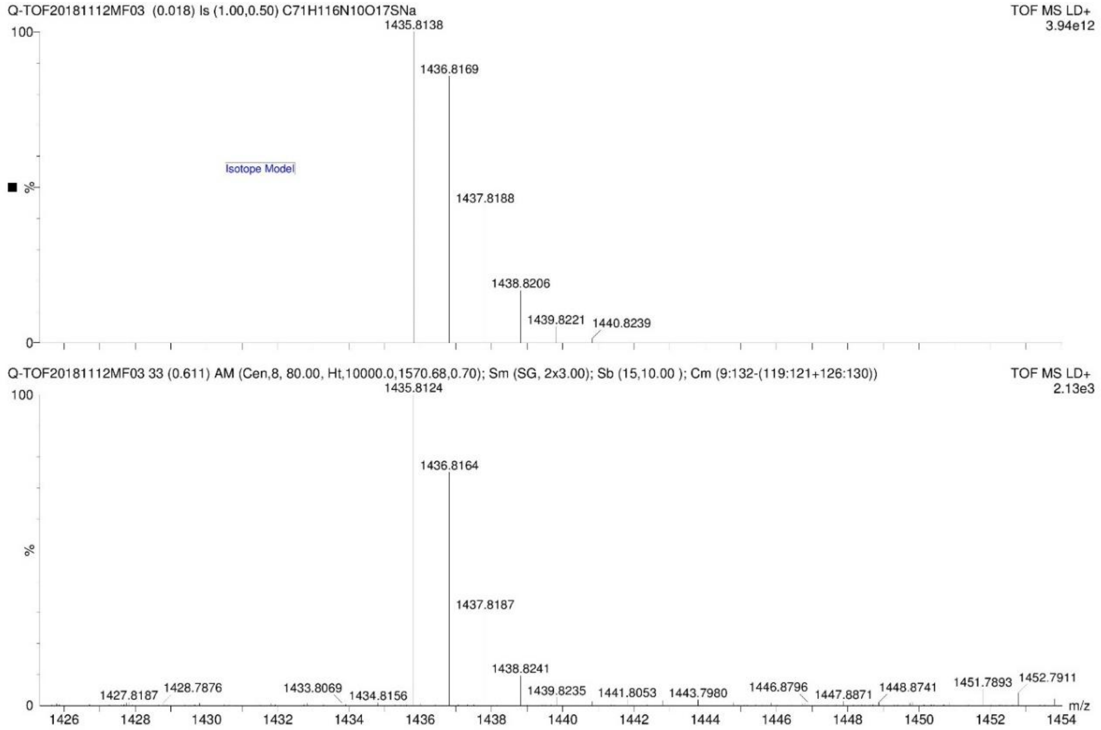

Supplementary Figure 23. Predicted isotopic model of peptide **1** (top) with MALDI high resolution mass spectrum of peptide **1** [M + Na]$^+$ showing the isotopic distribution (bottom).

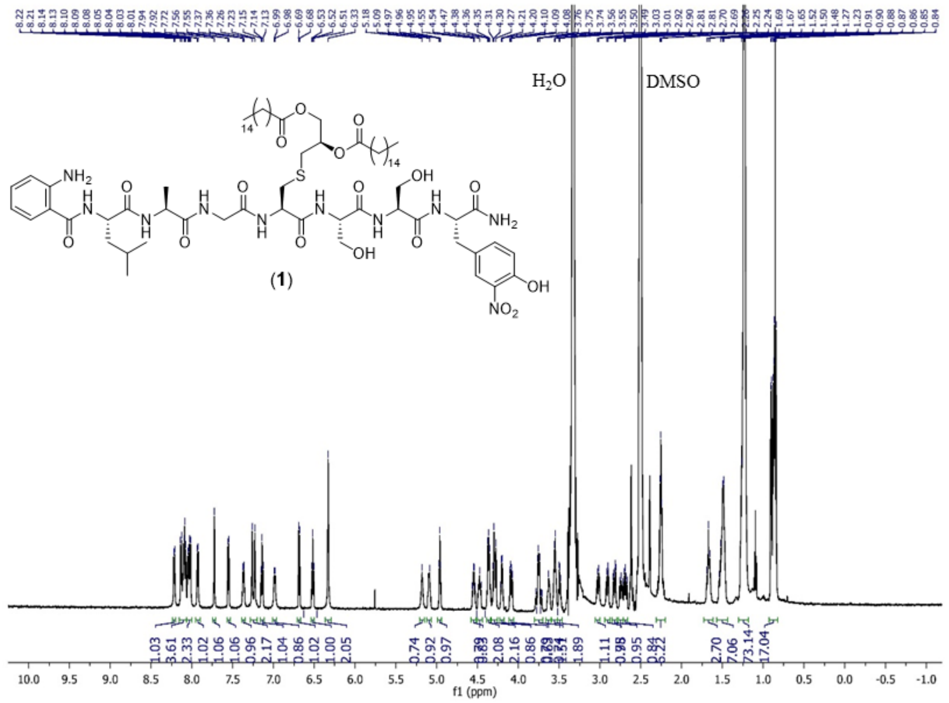

Supplementary Figure 24. ^{1}H NMR (600 MHz, DMSO-d$_6$) of peptide **1**

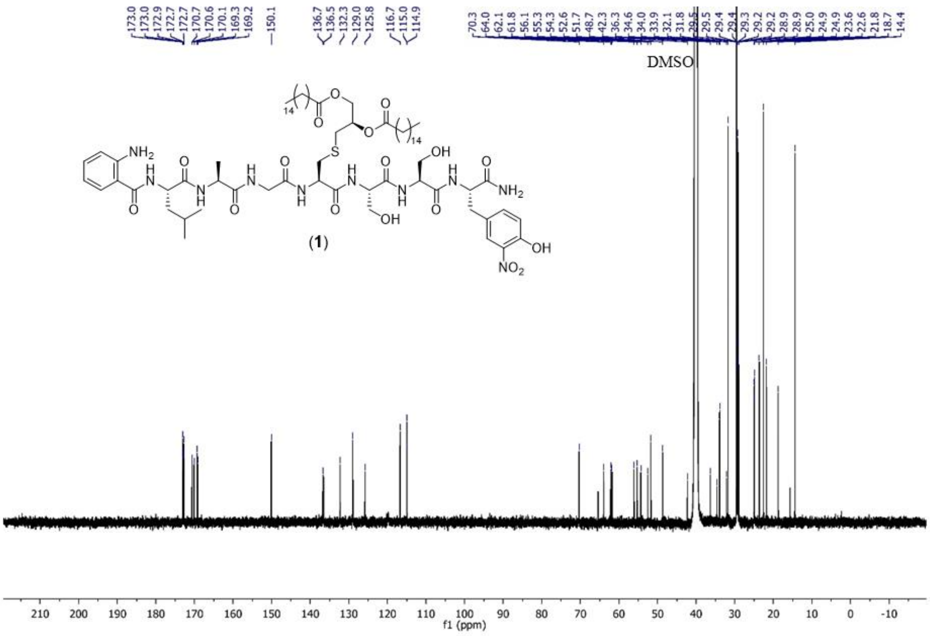

Supplementary Figure 25. ^{13}C NMR (151 MHz, DMSO-d_6) of peptide **1**

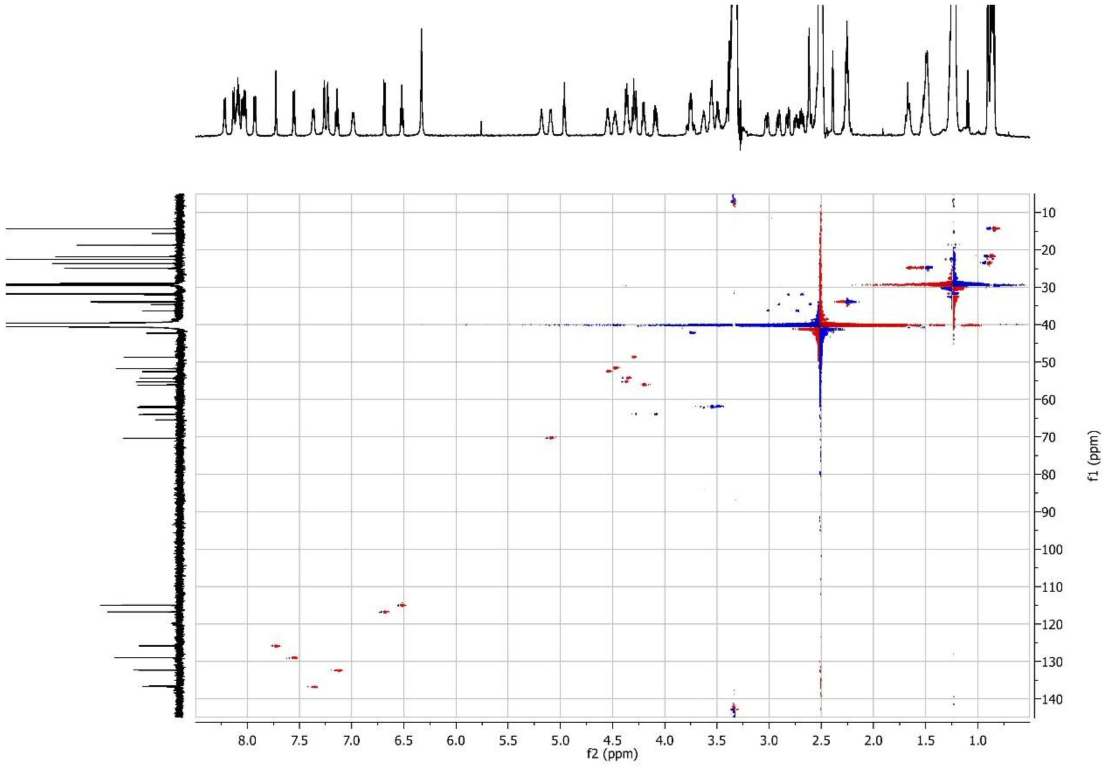

Supplementary Figure 26. HSQC (DMSO-d_6) of peptide **1**

Supplementary Tables

Supplementary Table 1: List of abbreviations

Abbreviation	Full name
Abz	aminobenzoic acid
DAG	diacylglyceryl
DOPG	1,2-dioleoylphosphatidylglycerol
EL	extracellular loop
FRET	fluorescence resonance energy transfer
G-P	glycerol-1-phosphate
H1-H4	transmembrane helices
ICP	inhibitor of cysteine protease
LCP	lipid cubic phase
Lgt	prolipoprotein diacylglyceryl transferase
Lnt	apolipoprotein N-acyltransferase
LspA	lipoprotein signal peptidase II
LspMrs	lipoprotein signal peptidase II from methicillin-resistant *Staphylococcus aureus*
LspPae	lipoprotein signal peptidase II from *Pseudomonas aeruginosa*
MRSA	methicillin-resistant *Staphylococcus aureus*
MSSA	methicillin-sensitive *Staphylococcus aureus*
PE	phosphatidylethanolamine
PG	phosphatidylglycerol
SP	signal peptide
TEV	tobacco etch virus
TEVp	tobacco etch virus protease

Supplementary Table 2. Specific activity of LspMrs and LspPae determined in FRET-based and gel-shift assays

Specific activity	FRET assay[a]		Gel-shift assay[b]	
	LspMrs	LspPae	LspMrs	LspPae
V (nmol/(mg.min))	1.62	87.52	31.2	90.6
k_{cat} ($10^{-3}.s^{-1}$)	0.50	30.86	9.55	31.96

[a] The values for LspMrs are based on initial reaction rates measured using an enzyme concentration of 0.3 µM and a substrate concentration of 80 µM. The corresponding values for LspPae were 0.1 µM and 30 µM.

[b] For both orthologs, the specific activities were measured using an enzyme concentration of 0.5 µM. The pre-proICP substrate concentration in the assay was 12 µM.

Supplementary Table 3. IC_{50} values for globomycin and myxovirescin determined in FRET-based and gel-shift assays of peptidase activity in wild-type LspPae and LspMrs

Construct	IC_{50} (µM)[a]			
	Gel-shift assay[b]		FRET assay[c]	
	Globomycin	Myxovirescin	Globomycin	Myxovirescin
LspPae	0.64	1.09	0.057	0.053
LspMrs	170.7	0.16	0.167	0.151

[a] The values shown correspond to the average of duplicate independent measurements.

[b] IC_{50} values are based on ICP product formed after 30 minutes of reaction. Assays were performed using an enzyme concentration of 0.5 µM. The pre-proICP substrate concentration in the assay was 12 µM.

[c] The values for LspMrs are based on initial reaction rates measured using an enzyme concentration of 0.3 µM and a substrate concentration of 80 µM. The corresponding values for LspPae were 0.1 µM and 30 µM.

Source data are provided as a Source Data file.

Supplementary Table 4. Contacts involving globomycin and myxovirescin in the LspMrs-globomycin and LspMrs-myxovirescin complex structures[a, b]

LspMrs-Globomycin

Partner 1	Partner 2	Distance (Å)
**g.Ser:O**	**Arg110:NH2**	**2.9**
**g.Ser:O**	**Asn52:OD1**	**3.7**
**g.Ser:O**	**Asn52:ND2**	**3.9**
**g.Ser:OG**	**Asn106:OD1**	**3.9**
**g.Ser:OG**	**Asp118:OD1**	**2.8**
**g.Ser:OG**	**Asn133:ND2**	**3.2**
**g.Ser:OG**	**Asn133:OD1**	**3.9**
**g.Ser:OG**	**Asp136:OD2**	**2.4**
**g.Ser:OG**	**Asp136:OD1**	**3.0**
**g.Ser:N**	**Asp136:OD1**	**3.0**
**g.Ser:N**	**Asn106:ND2**	**3.6**
**g.Ser:N**	**Arg110:NH2**	**3.8**
**g.Ile:O**	**Arg110:NH2**	**2.3**
**g.Ile:O**	**Arg110:NH1**	**3.9**
**g.Leu:O**	**Arg110:NH1**	**3.7**
**g.Leu:O**	**Asn106:ND2**	**2.9**
g.Gly:O	Trp57:NE1	4.1
**g.Gly:N**	**Arg110:NH2**	**3.3**
g.Gly:N	g.Ile:O	3.0
g.Thr:O	Trp57:NE1	2.8
**g.Thr:O**	**Gly54:N**	**4.0**
**g.Thr:OG**	**Asn52:ND2**	**3.0**
**g.Thr:OG**	**Asn52:OD1**	**3.8**
**g.Thr:N**	**Asn52:ND2**	**3.3**
g.Thr:OG	OLC4:O23	2.4
g.Thr:N	OLC4:O23	2.8

LspMrs-Myxovirescin

Partner 1	Partner 2	Distance (Å)
**m.CO6:O42**	**Asp118:OD1**	**3.2**
**m.CO6:O42**	**Asp118:OD2**	**2.6**
**m.CO6:O42**	**Asn133:ND2**	**3.3**
**m.CO6:O42**	**Asp136:OD2**	**2.7**
**m.CO6:O42**	**Asp136:OD1**	**3.4**
**m.NO4**	**Asp136:OD1**	**2.7**
**m.NO4**	**Asn106:ND2**	**3.3**
**m.NO4**	**Arg110:NH2**	**3.7**
**m.CO3:O29**	**Arg110:NH2**	**3.0**
m.CO3:O29	Ala56:NH2	3.5
**m.CO3:O29**	**Gly54:O**	**4.1**
**m.C28:O31**	**Asn106:ND2**	**3.5**
**m.C28:O31**	**Arg110:NH1**	**3.2**
**m.C28:O31**	**Arg110:NH2**	**3.8**
m.CO8:O41	Ile131:O	2.9
**m.CO8:O41**	**Asp118:OD1**	**2.5**
**m.CO8:O41**	**Asp118:OD2**	**3.8**
**m.CO8:O41**	**Asn52:ND2**	**3.1**
m.CO9:O40	Trp57:N	2.9
m.CO9:O40	Ala55:O	4.1
m.C20:O30	Ala56:NH2	3.7

[a] Antibiotic atoms that overlap in the complex structures (referred to as spine atoms) are highlighted in grey.

[b] Entries in bold refer to contacts with highly conserved residues.

Supplementary Table 5. Primers used for mutagenesis

Name	Sequence[a]
N52A_F[b]	CAGCCACCGTGCCAATGGCGCGG
N52A_R[b]	GTAATGTTCAGAAAATGCGGGATC
N52Q_F	CAGCCACCGTCAGAATGGCGCGG
N52Q_R	GTAATGTTCAGAAAATGCGGGATCAC
G54A_F	CCGTAACAATGCAGCGGCCTGGG
G54A/P_R	TGGCTGGTAATGTTCAGAAAATG
G54P_F	CCGTAACAATCCGGCGGCCTGGG
G54A/P_R	TGGCTGGTAATGTTCAGAAAATG
R110A_F	TTTTATTGACGCCGTTCTGACCGGTGAAGTGGTTG
R110A/K_R	TTACCCAGAGCGCCTGCG
R110K_F	TTTTATTGACAAAGTTCTGACCGGTGAAGTGGTTGATTTCATCG
D118N_F	TGAAGTGGTTAACTTCATCGACACGAAC
D118N_R	CCGGTCAGAACGCGGTCA
N133A_F	CCCGATCTTTGCCATTGCCGACAG
N133A/Q_R	AAATCATAGCCAAAAATGTTC
N133Q_F	CCCGATCTTTCAAATTGCCGACAG
D136N_F	TAATATTGCCAACAGCTCTCTGAC
D136N_R	AAGATCGGGAAATCATAGC

[a] Primer sequences are from the 5’ end to the 3’ end.
[b] ‘F’ and ‘R’ refer to the forward and reverse amplification primers, respectively.

Supplementary References

1. Vogeley, L. *et al.* Structural basis of lipoprotein signal peptidase II action and inhibition by the antibiotic globomycin. *Science* **351**, 876–80 (2016).

2. Epand, R. F., Savage, P. B. & Epand, R. M. Bacterial lipid composition and the antimicrobial efficacy of cationic steroid compounds (Ceragenins). *Biochim. Biophys. Acta - Biomembr.* **1768**, 2500–2509 (2007).

3. Kitamura, S. & Wolan, D. W. Probing substrate recognition of bacterial lipoprotein signal peptidase using FRET reporters. *FEBS Lett.* **592**, 2289–2296 (2018).

4. Segel, I. H. *Enzyme Kinetics*. (Wiley, New York, 1975).

5. Copeland, R. A. *Enzymes: a practical introduction to structure, mechanism, and data analysis*. (Wiley-VCH, 2000).

6. Holdgate, G. A., Meek, T. D. & Grimley, R. L. Mechanistic enzymology in drug discovery: a fresh perspective. *Nat. Rev. Drug Discov.* **17**, 115–132 (2018).

7. Dev, I. K., Harvey, R. J. & Ray, P. H. Inhibition of prolipoprotein signal peptidase by globomycin. *J. Biol. Chem.* **260**, 5891–5894 (1985).

8. Tokunaga, M., Loranger, J. M. & Wu, H. C. Prolipoprotein modification and processing enzymes in Escherichia coli. *J. Biol. Chem.* **259**, 3825–3830 (1984).

9. Kitamura, S., Owensby, A., Wall, D. & Wolan, D. W. Lipoprotein Signal Peptidase Inhibitors with Antibiotic Properties Identified through Design of a Robust In Vitro HT Platform. *Cell Chem. Biol.* **25**, 301-308.e12 (2018).

10. Morrison, J. F. Kinetics of the reversible inhibition of enzyme-catalysed reactions by tight-binding inhibitors. *BBA - Enzymol.* **185**, 269–286 (1969).

11. Reichel, F. *et al.* Stereochemical dependence of the self-assembly of the immunoadjuvants Pam3Cys and Pam3Cys-Ser. *J. Am. Chem. Soc.* **121**, 7989–7997 (1999).

12. Niederhafner, P., Šafarík, M., Brichtová, E. & Šebestík, J. Rapid acidolysis of benzyl group as a suitable approach for syntheses of peptides naturally produced by oxidative stress and containing 3-nitrotyrosine. *Amino Acids* **48**, 1087–1098 (2016).

13. Bromfield, K. M., Cianci, J. & Duggan, P. J. The preparation of fluorescence-quenched probes for use in the characterization of human factor Xa substrate binding domains. *Molecules* **9**, 427–439 (2004).

14. Nair, D. *et al.* Whole-genome sequencing of Staphylococcus aureus strain RN4220, a key laboratory strain used in virulence research, identifies mutations that affect not only virulence factors but also the fitness of the strain. *J. Bacteriol.* **193**, 2332–2335 (2011).

15. Landau, M. *et al.* ConSurf 2005: the projection of evolutionary conservation scores of residues on protein structures. *Nucleic Acids Res.* **33**, 299–302 (2005).

16. Liebschner, D. *et al.* Polder maps: improving OMIT maps by excluding bulk solvent. *Acta Crystallogr. Sect. D, Struct. Biol.* **73**, 148–157 (2017).
